# Unravelling recent advances in 1,2,4-triazole-based anticancer agents: synthetic strategies, molecular targets, molecular docking studies, and structure–activity relationships

**DOI:** 10.1039/d6ra05942b

**Published:** 2026-07-31

**Authors:** Glanish Jude Martis, Eshitha Jane D Souza, Praveen S. Mugali, Santosh L. Gaonkar

**Affiliations:** a Manipal Institute of Technology, Manipal Academy of Higher Education Manipal India sl.gaonkar@manipal.edu; b Department of U. G. Studies in Chemistry, Alva's College (Autonomous) Moodubidire, Dakshina Kannada Karnataka 574227 India; c Department of P. G. Studies in Chemistry, Alva's College (Autonomous) Moodubidire, Dakshina Kannada Karnataka 574227 India

## Abstract

1,2,4-Triazoles have been interesting in the field of heterocyclic chemistry and their utility has been expanded to biological areas including cancer therapy, drug research and development. Thus, this review focuses on the synthetic aspects and biological role played by 1,2,4-triazoles in anticancer treatment and research. Epidermal growth factor receptors (EGFRs), vascular endothelial growth factor-2 (VEGFR-2) and carbonic anhydrases are important classes of oncogenic targets that have attracted significant attention because of their role in cancer studies. By inhibiting these target-specific enzymes it is possible to treat various types of cancer for which 1,2,4-triazoles have shown tremendous progress in development as anticancer agents. Therefore, this review presents recent advances in 1,2,4-triazole chemistry and its biological role in the development of anticancer targets through the inhibition of EGFR, VEGFR-2 and different carbonic anhydrase enzymes. Synthetic aspects help us understand the importance of molecular generation in drug design and discovery. Anticancer studies are helpful for determining the types of active targets among the class of 1,2,4-triazoles. Molecular docking studies reveal major interactions with several amino acid residues and furnish better comparisons with standard reference drugs. A structure–activity relationship (SAR) study enables us to determine the role played by the substituents linked to 1,2,4-triazole hybrids. Herein, we discuss the latest advances in the identification of 1,2,4-triazole-based anticancer targets and active compounds, with the exclusive literature covering the last six years.

## Abstract 2

1,2,4-Triazoles have been interesting in the field of heterocyclic chemistry and their utility has been expanded to biological areas including cancer therapy, drug research and development.

## Graphical abstract

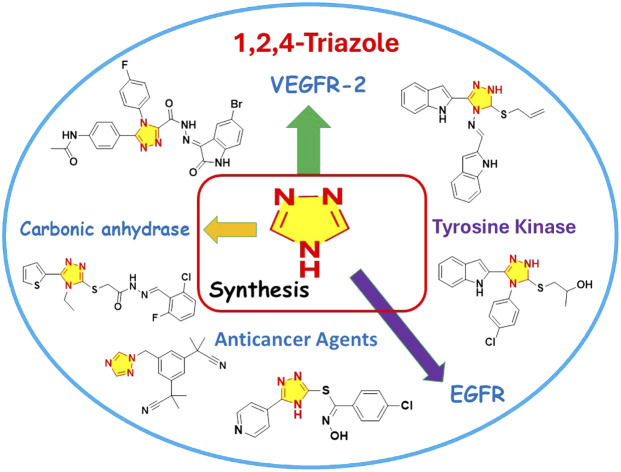

## 1 Introduction

**Fig. 1:**
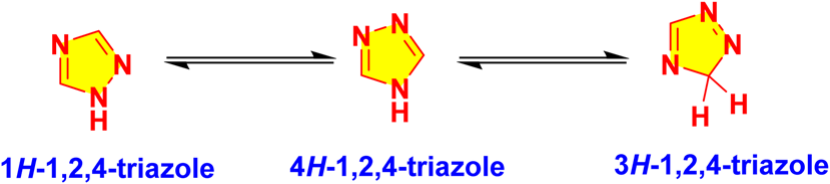
Isomeric forms of 1,2,4-triazole.

**Fig. 2:**
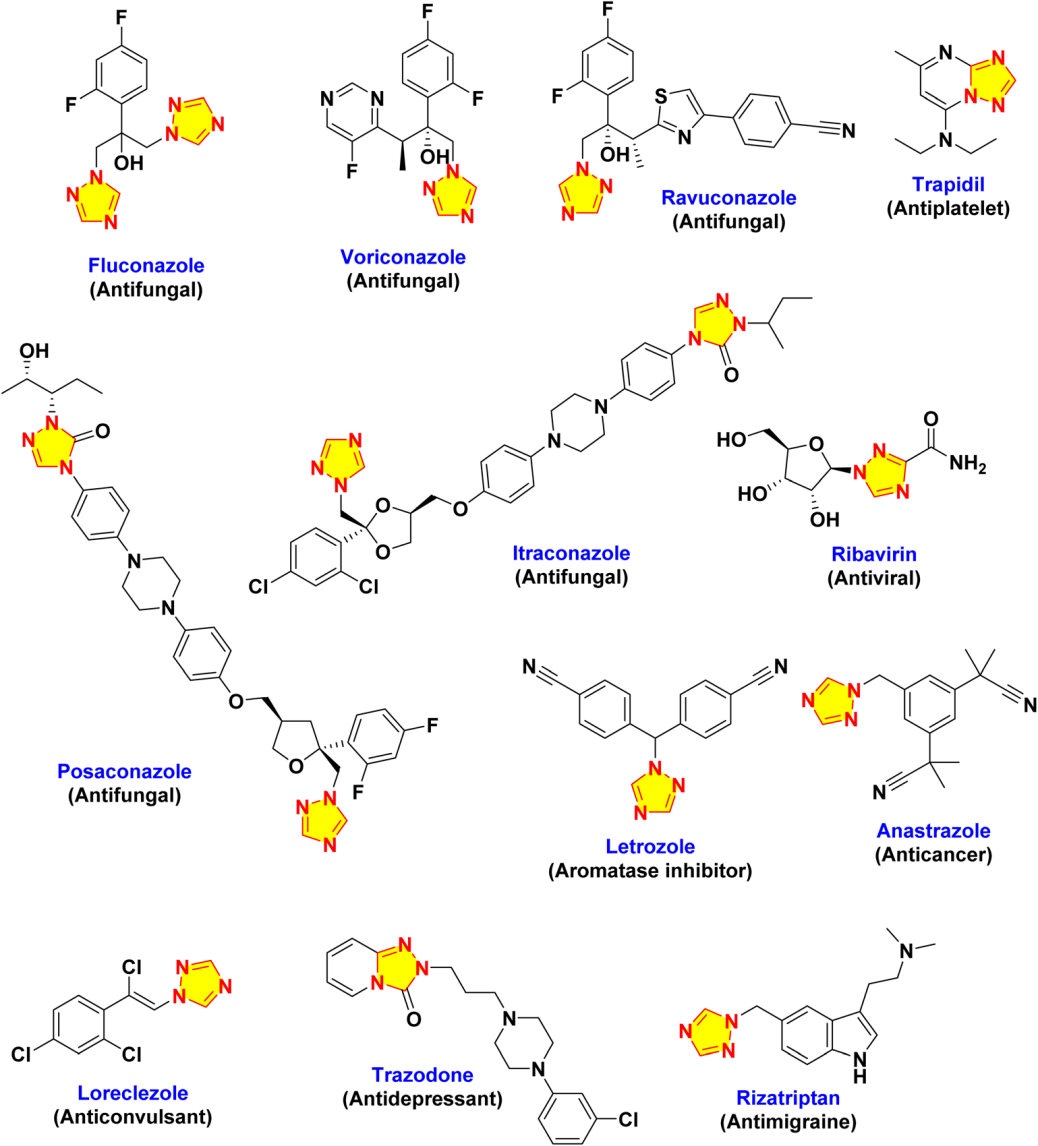
1,2,4-Triazole-containing FDA approved drug molecules.

For many years, heterocyclic compounds have created their own path in the field of synthetic and medicinal chemistry.^1–7^ Ultimately, the biological and pharmacological performance of azoles has become promising. Therefore, their use in the field of medicinal chemistry is much more demanding and new doors of innovation should be opened.^8–12^ 1,2,4-Triazoles have been useful in terms of their potential as anticancer agents which exist in three isomeric forms (Fig. 1).^13–19^ There are many drug molecules containing 1,2,4-triazoles. A few of them include fluconazole,^20–22^ posaconazole,^23–25^ voriconazole,^26–28^ itraconazole,^29–31^ ravuconazole^32–34^ (antifungals), ribavirin^35,36^ (antiviral), letrozole^37,38^ (aromatase inhibitor), anastrazole^39,40^ (anticancer), loreclezole^41–43^ (anticonvulsant), trazodone^44–46^ (antidepressant), trapidil^47,48^ (antiplatelet), rizatriptan^49–51^ (antimigraine) and many more (Fig. 2).

Epidermal growth factor receptors (EGFRs) are tyrosine kinases that are also known as HER1 or ErbB1. Tyrosine kinases are widely known for their ability to regulate many cellular activities during cell growth, division and metabolism.^52–54^ The transfer of phosphate groups from ATP to other tyrosine residues within proteins is regulated by these enzymes. It is possible to combat the rapid division of cancer cells and the risk of cancer in humans by inhibiting EGFR kinases.^55–58^ EGFR pathways are not limited to the above cases, rather they are also involved in the regulation of cell proliferation, survival and migration.^59,60^ Prevention of downstream signaling is very much necessary and for this to happen new organic molecules should be developed in the coming years. EGFR inhibitors are chosen to prevent cancer because of their key role in cell signaling pathways including cell proliferation, apoptosis, angiogenesis and metastatic spread.^61–69^ Therefore, chemists need to develop new molecules such as 1,2,4-triazole hybrids that have been proven to be crucial for the development of anticancer agents.

Vasculogenesis and angiogenesis are the two major processes that occur with the help of vascular endothelial growth factor (VEGF).^70–73^ The human body is a multiorgan functional system that regulates balance and creates imbalances because of several factors such as mutations and other anomalies that occur within the body. Several processes regulate cell proliferation, and numerous molecules play vital roles in maintaining bodily functions.^74–77^ Eventually, when proangiogenic and antiangiogenic molecules enter a state of imbalance, the level of VEGF increases, triggering the activation of tumor cells. VEGFR-1 (fetal liver kinase 1), VEGFR-2 (kinase insert domain receptor) and VEGFR-3 (fetal liver kinase 4) are the three main subtypes of VEGF.^78–81^

1,2,4-Triazoles act as competitive inhibitors disrupting ATP-binding pockets of tyrosine kinases such as EGFR and VEGFR-2 by mimicking the adenine ring of ATP *via* H-bonding which projects hydrophobic tails to the adjacent allosteric sites. The three nitrogen atoms in 1,2,4-triazoles act as hydrogen bond acceptors and donors similar to natural adenine ring of ATP with hinge interactions. ATP-pocket disruption is based on the rigidity and planarity of 1,2,4-triazole core structure fitting snugly into the flat-front of ATP-binding cleft which prevents the entry of natural ATP or phosphorylation of downstream targets.^82,83^

Cancer cell apoptosis is triggered by 1,2,4-triazole derivatives by modulating Bcl-2 proteins and arresting cells at G1/S or G2/M checkpoints. This results in the inhibition of tyrosine kinases by lowering mitochondrial membrane potential resulting into the release of cytochrome c into the cytosol. Depending on the specific cancer cell lines and molecular targets, these derivatives block the progression at G1 phase, S phase, or G2/M phase.^84,85^

Focusing on the structure of 1,2,4-triazole, it renders high dipole moment with balanced H-bonding capacity. The strong local dipole moment offered by the three nitrogen atoms of 1,2,4-triazole prevents excessive lipophilicity, in turn preventing trapping of compounds. Water solubility is significantly boosted due to electrostatic charge distribution paving a way for better formulation and their systemic distribution in bodily fluids. This results into enhancement of metabolic stability, aqueous solubility, and target binding affinity. Such physicochemical properties lead to the improvement of membrane permeability and reduction in enzymatic degradation in turn optimizing pharmacokinetic profiles of anticancer drugs.^86–89^

Carbonic anhydrases are important zinc-based metallozymes that are involved in the conversion of carbon dioxide and water into bicarbonate as well as into hydrogen ions and *vice versa*.^90,91^ This reversible reaction is mediated by carbonic anhydrases which trigger rapid proliferation of tumor cells; therefore, their inhibition and control are necessary.^92,93^ Apart from their negative effects, they are also useful in several processes including pH regulation, respiratory processes, gas transport, and digestion in the stomach because they produce hydrochloric acid.^94,95^ Despite having many merits, these enzymes have been fatal in many cases and need to be addressed frequently by in-depth research in the field of chemistry and biology.^96–98^

In our previous work, we discussed the recent advances in the use of 1,2,3-triazoles with the goal of understanding their synthetic aspects and overall biological prospects.^99^ Additionally, recent advances in 1,2,4-triazoles highlighting the importance of inhibition of kinases, carbonic anhydrases, topoisomerases is widely studied.^84^ In this review, we highlight the importance of 1,2,4-triazole focused exclusively on anticancer activity. The field of anticancer research is vast; therefore, our focus was exclusively on three main oncogenic targets namely, EGFR, VEGFR-2 and carbonic anhydrase inhibitors. This review presents the latest advances in the development of 1,2,4-triazole-containing hybrids with anticancer profiles with detailed discussion based on the literature covering the past six years. Therefore, this review is more helpful for researchers and scientists working in the field of medicinal chemistry and triazole chemistry.

## 2 Synthetic developments of 1,2,4-triazole hybrids

**Fig. 3:**
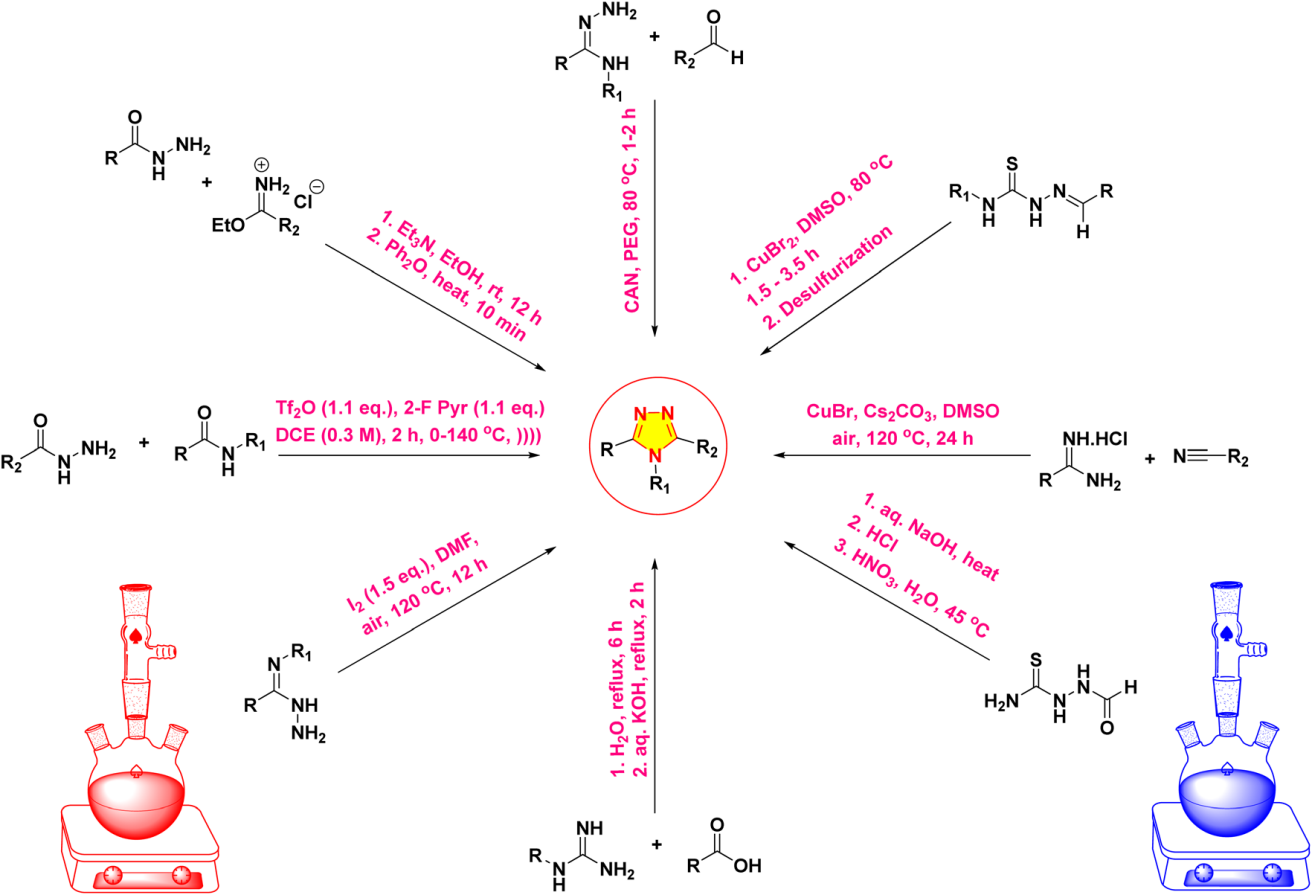
General methods for the synthesis of 1,2,4-triazoles.

1,2,4-Triazoles have been synthesized since many years for the development of new medicinally active compounds. They are generally produced from classical methods and emerging new methods of synthesis. They are often produced from the reaction of various hydrazides with substituted amides, iodine-facilitated acetamidohydrazides reactions, reaction of guanidine substrates with various carboxylic acids, substituted hydrazones, cyclization of 2-formylhydrazine-1-carbothioamide, reaction of acetimidamide hydrochlorides with different nitriles, copper-catalyzed thiosemicarbazone desulfurization, CAN and PEG mediated reaction between substituted hydrazonamides and aldehydes (Fig. 3).^13^

Now we discuss different synthetic routes for the production of 1,2,4-triazole scaffolds with good EGFR, VEGFR-2 and carbonic anhydrase inhibition with their role in anticancer activity.

### 2.1 EGFR kinase inhibitors

**Scheme 1:**
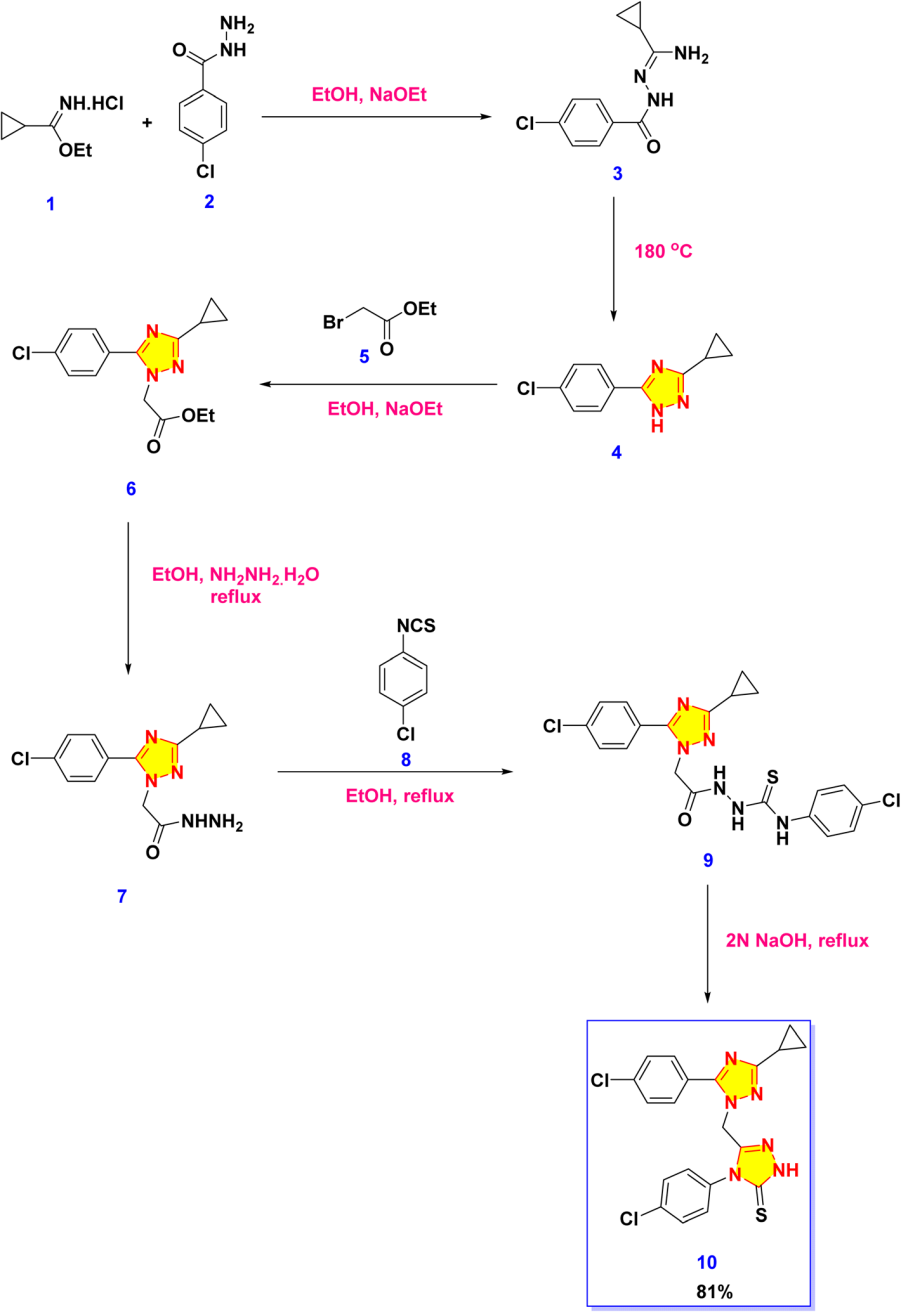
Synthesis of 1,2,4-triazole-1,2,4-triazole-3-thione 10.

1,2,4-Triazoles when clubbed with 1,2,4-triazole-3-thione moiety, showed increased EGFR inhibition activity for which there was significant contributions from both the core parts. Kolcuoglu and coworkers described a method for synthesizing 1,2,4-triazole scaffold 10 starting from ethyl cyclopropanecarbimidate hydrochloride 1 and 4-chlorobenzohydrazide 2 to give intermediate 3 which upon heating at 180 °C produced 1,2,4-triazole ring 4. *N*-alkylation of triazole ring 4 was carried out using metallic sodium in absolute ethanol and later treated with ethyl bromoacetate 5 followed by hydrazide 7 formation using hydrazine hydrate. The hydrazide part was further extended to thiosemicarbazide moiety 9 which was essential for the formation of 1,2,4-triazole-3-thione 10 core part using 2N sodium hydroxide. This compound 10 showed an IC_50_ value of 0.4 µM against EGFR kinase. Interestingly, it had prominent interactions with several amino acid residues which resembled with standard EGFR kinase inhibitor Gefitinib. These include, formation of two H-bonds by the 1,2,4-triazole-3-thione ring with Met793 and 1*H*-1,2,4-triazole formed one H-bond with Cys797. Alongside, the cyclopropyl substitution on the triazole ring offered hydrophobic interaction with Gly796. The chlorine atom on the phenyl groups acted as sidechain acceptor for Lys745. Furthermore, the phenyl ring contributed with arene–H interactions with Val726 (Scheme 1).^100^

**Scheme 2:**
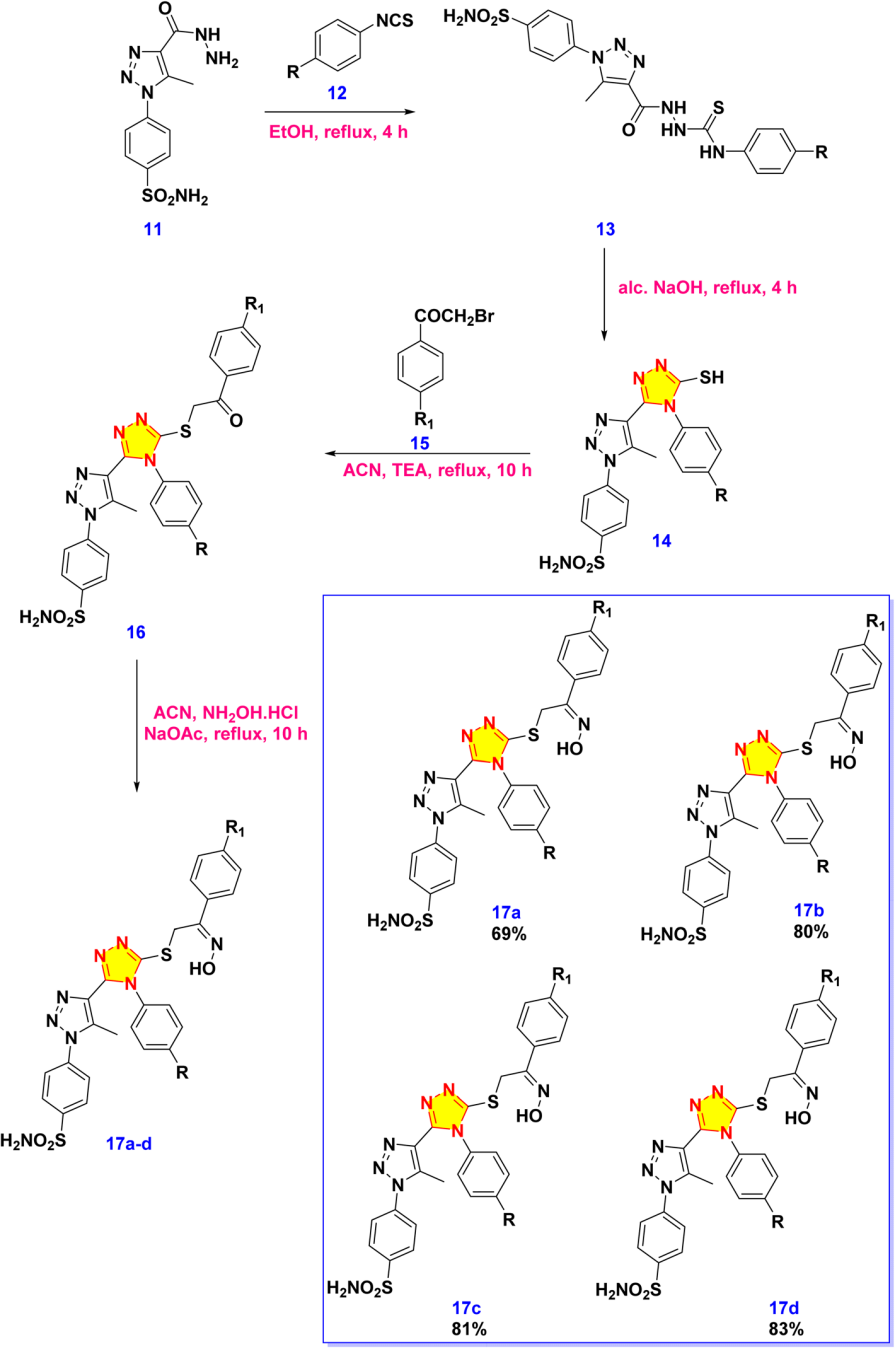
Synthesis of 1,2,3-triazole and 1,2,4-triazole sulfonamide-based aromatic EGFR inhibitors 17a–d.

1,2,4-Triazoles when linked with 1,2,3-triazoles with oxime formation 17a–d, exhibited potent activity against EGFR kinases and other enzymes. 1,2,3-Triazole-hydrazides 11 were extended to thiosemicarbazide moiety 13 using different phenyl isothiocyanates 12 and later cyclized to 1,2,4-triazole-5-thiol 14 using alcoholic NaOH. This thiol 14 was further acylated using different phenacyl bromide derivatives 15. The phenacyl parts were then converted to oxime using hydroxylamine hydrochloride in the presence of acetonitrile and sodium acetate. Compounds 17a–d significantly inhibited the activity of EGFR kinase with IC_50_ values of 0.112, 0.205, 0.169, 0.066 µM, respectively. Additionally, these compounds also showed promising activity in inhibiting A549 cancer cells with IC_50_ values of 4.5, 3.0, 4.0 and 3.3 µM compared with Sorafenib. The sulfonamide moiety directly linked to 1,2,3-triazole acts as a privileged scaffold which is in turn attached to 1,2,4-triazole is responsible for enhanced EGFR kinase inhibition. This dual triazole moieties improved cytotoxicity with good IC_50_ values as described above. Molecular docking studies revealed that the docking scores of −8.7, −8.9, −8.8 and −8.8 Kcal mol^−1^ against EGFR kinase compared with encorafenib and 4-4-anilinoquinazoline were closely associated. However, these docking scores were further supported with several molecular interactions such as three H-bonds with Val702, Met769, Lys721 through triazole rings and –SO_2_NH_2_ moieties. Alongside, π–π stacking with Asp830, Lys721, Met769, and Thr830 and many more as listed in the Table 1 and (Scheme 2).^101^

**Table 1:** Overview of 1,2,4-triazole-containing EGFR inhibitors

Compounds	Substituents	Major interactions	SAR observations
10	4-Chlorophenyl groups attached to 1,2,4-triazole and 1,2,4-triazole-3-thione moiety. Cyclopropyl ring linked to 1,2,4-triazole	1,2,4-Triazole-3-thione C=S forms two H-bonds with Met793 and triazole ring forms one H-bond with Cys797. Cyclopropyl group offers hydrophobic contact with Gly796	Electron-withdrawing chlorophenyl group improved cytotoxicity
17a	4-Phenyl and 4-anisyl groups attached to 1,2,4-triazole rings	Three H-bonds with Val702, Met769, Lys721. π–π stacking with Asp830, Lys721, Met769, and Thr830	Electron pulling effect rendered by phenyl ring and electron-donating capacity offered by anisyl group increased the EGFR kinase inhibition activity
17b	4-Phenyl and 4-nitrophenyl rings attached to 1,2,4-triazole moiety	Three H-bonds with Val702, Met769, Lys721. π–π stacking with Lys721, Lys773, Met742, Asp831	Electron-withdrawing effect contributed from aromatic ring systems activates triazole ring for the enhanced biological performance
17c	4-Anisyl and 4-tolyl rings attached to 1,2,4-triazole ring	Three H-bonds with Val702, Met769, Lys721. π–π stacking with Lys721, Leu694, Gly772, Cys773 and Met769	Both electron-donating anisyl and tolyl groups offer good hydrophobic character to be developed for the enhanced activity
17d	4-Anisyl and 4-nitrophenyl linked to 1,2,4-triazole ring	Three H-bonds with Val702, Met769, Lys721. π–π stacking with Pro770, Lys721, Cys773, Met742	Both electron-donating and electron-withdrawing groups offer push–pull effect
23	4-Chlorophenyl linked to 1,2,4-triazole oxime	H-bonding with Glu738, Gln767, Leu694 and π–H interaction with Leu694	Phenyl ring with the electron-withdrawing chloro group interacts with the active site of EGFR and increases inhibition activity
32	4-Chlorophenyl linked to 1,2,3-triazole-fused 1,2,4-triazolothiazine	H-bonding with Phe699, Gly695, Gly700. Electrostatic interaction with Lys721. Hydrophobic contacts with Val702	Phenyl rings with electron-withdrawing nitro and chloro groups tend to increase the EGFR kinase inhibition activity
33	3,5-Chlorophenyl linked to 1,2,3-triazole-fused 1,2,4-triazolothiazine	H-bonding with Lys721. Electrostatic interaction with Lys704. Hydrophobic interaction with Val702	Two electron-withdrawing chlorine atoms significantly enhance EGFR kinase inhibition activity
41a	4-Fluorophenyl ring attached to 1,2,3-triazole connected 1,2,4-triazole	H-bond with Cys773 and π–π stacking with Asp831, Met857, Ala835, Leu838, Tyr845, Phe699	Electronegative fluorine atom drawn electron pull effect which activates the phenyl ring to increase anticancer effect
41b	3,5-Dichlorophenyl ring attached to 1,2,3-triazole connected 1,2,4-triazole	H-bond with Asn818 and π–π stacking with Asp831, Met857, Ala835, Leu838, Tyr845, Phe699	Two electron-withdrawing chlorine atoms significantly enhance EGFR kinase inhibition activity
46	3,4-Dimethoxyphenyl ring attached to chalcone embedded 1,2,4-triazole hybrid	H-bonding with Met769. Electrostatic interactions with Asp776, Lys721, Thr766, Cys773. Hydrophobic contacts with Ala719, Phe832, Leu820	Electron-donating methoxy groups attached to the phenyl ring contributed for the EGFR inhibition by occupying binding site of EGFR kinase
51	Phenyl ring directly substituted to 1,2,4-triazole	H-bonding with Met793 and one arene–cation interaction with Lys745	Electron rich phenyl aromatic ring activates the triazole ring and enhances the activity

**Scheme 3:**
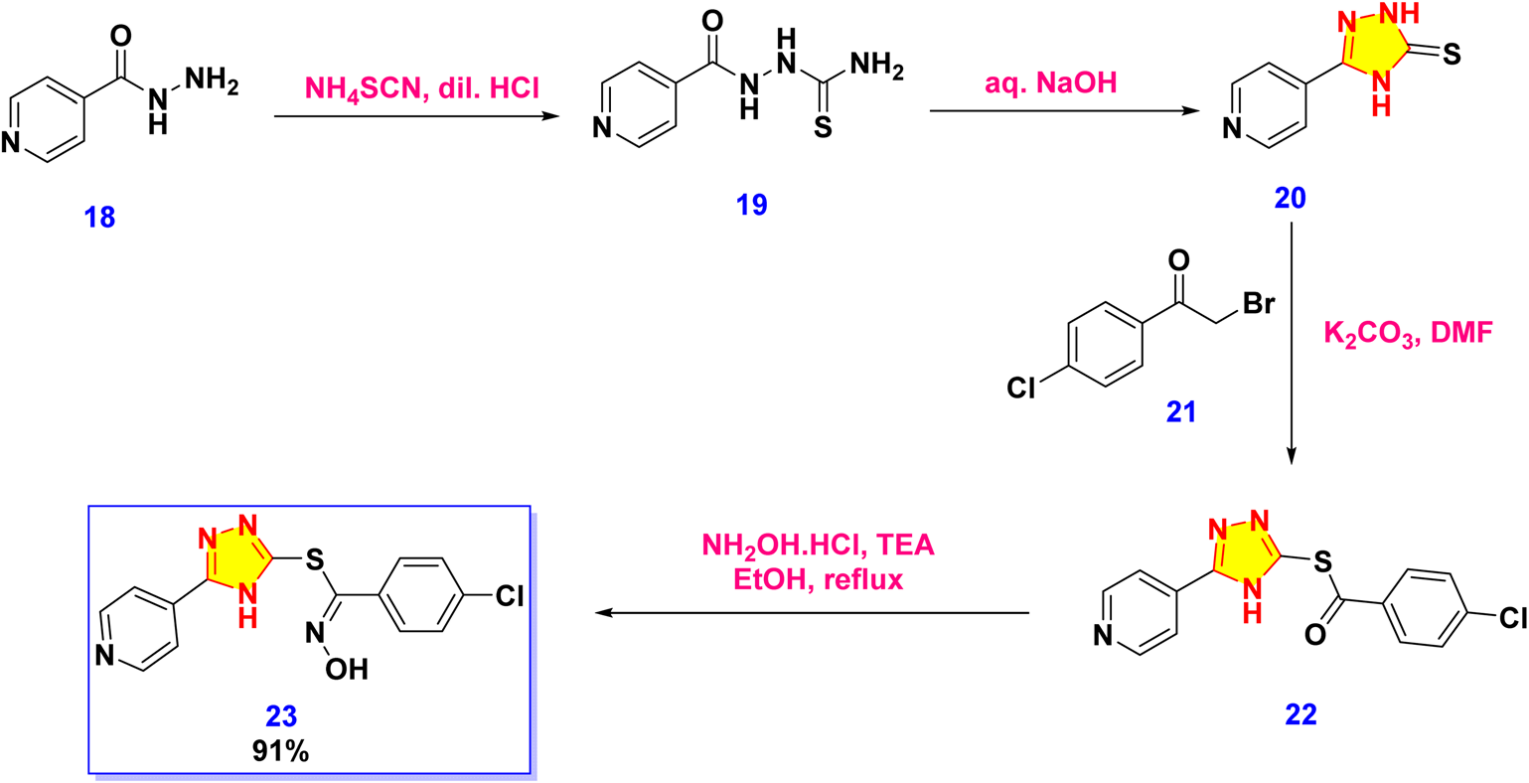
Synthesis of pyridine-containing 1,2,4-triazole oxime EGFR inhibitor 23.

Oximes were influential in inhibiting EGFR kinases when they are combined with pyridine-containing 1,2,4-triazole compounds. Likewise, compound 23 displayed good activity in inhibiting EGFR tyrosine kinase and other cancer cells. The synthesis of this compound 23 started from the treatment of ammonium thiocyanate to isoniazid 18 in the presence of dilute hydrochloric acid to give thiosemicarbazide derivative 19 which was cyclized to 1,2,4-triazole-3-thione intermediate 20 using aqueous sodium hydroxide. As we know, that this intermediate 20 undergoes keto-enol tautomerism and further it can be extended using acylation using 4-chlorophenacyl bromide 21 and the carbonyl group is transformed to target oxime of pyridine-containing 1,2,4-triazole 23. It showed an IC_50_ value of 0.18 ± 0.007 µM against EGFR kinase when compared with standard drug Gefitinib. Its binding energy was found to be −6.6 Kcal mol^−1^ in comparison with Erlotinib. Molecular docking studies showed prominent interactions of this target compound with four different amino acid residues such as including H-bonding with Glu738, Gln767, Leu694 and π–H interaction with Leu694 which had similar interactions with standard drug Erlotinib (Scheme 3).^102^

**Scheme 4:**
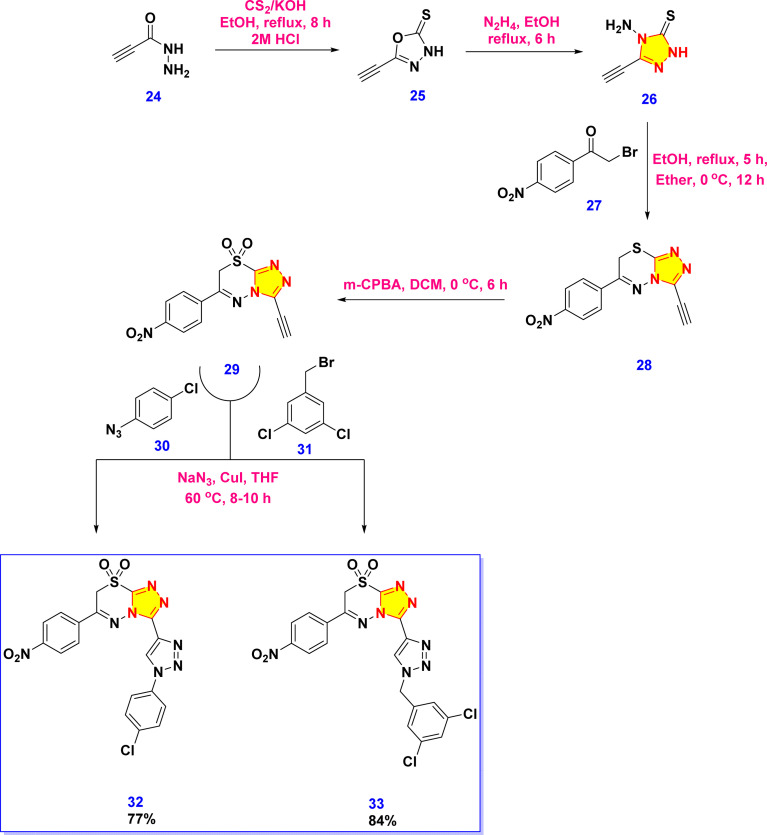
Synthesis of 1,2,4-triazolo-fused 1,4-thiazine compounds **32** and **33**.

Click chemistry also contributed a lot in the field of synthetic medicinal chemistry producing several 1,2,3-triazoles using 1,3-dipolar cycloaddition approach attributed by copper-azide cycloaddition. 1,2,4-Triazolo-fused compounds 32 and 33 have been seen successfully inhibiting EGFR kinase which were produced from cyclization of 5-ethynyl-1,3,4-oxadiazole-2(3*H*)-thione 25 from propiohydrazide 24 and its conversion to 4-amino-5-ethynyl-2,4-dihydro-3*H*-1,2,4-triazole-3-thione 26 using hydrazine and later cyclized to form fused-1,2,4-triazole-1,4-thiazine 28. Another key step in converting sulfur to sulfur dioxide 29 using *m*-CPBA in freezing conditions and diverts into two routes of producing target compounds 32 and 33 using Cu-catalyzed alkyne azide cycloaddition reacting *via* 4-chlorophenylazide 30 and 3,5-dichlorobenzylbromide 31. Compound 32 showed good activity against MCF-7, MDA-MB231 and MCF-10A cancer cells with IC_50_ values of 4.45 ± 0.35, 7.73 ± 0.44, 13.43 ± 0.85 µM. Also, compound 33 exhibited promising activity against the same cancer cells with IC_50_ values of 4.09 ± 0.25, 6.09 ± 0.58, 16.71 ± 0.93 µM. Both these compounds possess a direct linkage between 1,2,3-triazole and fused-1,2,4-triazole-1,4-thiazine which is responsible for enhanced activity with multi-target inhibition. Polar interactions and H-bonding with amino acid residues in both the ring systems offer increased EGFR kinase inhibition. 1,2,3-Triazole acts as a metabolic linker boosting aqueous solubility and cellular uptake whereas 1,2,4-triazole offers robust binding affinity. However, these two compounds had strong interactions like H-bonding with Phe699, Gly695 and Gly700. Moreover. Electrostatic interaction with Lys72, Lys704 and hydrophobic contacts with Val702 were commendable in docking studies (Scheme 4).^103^

**Scheme 5:**
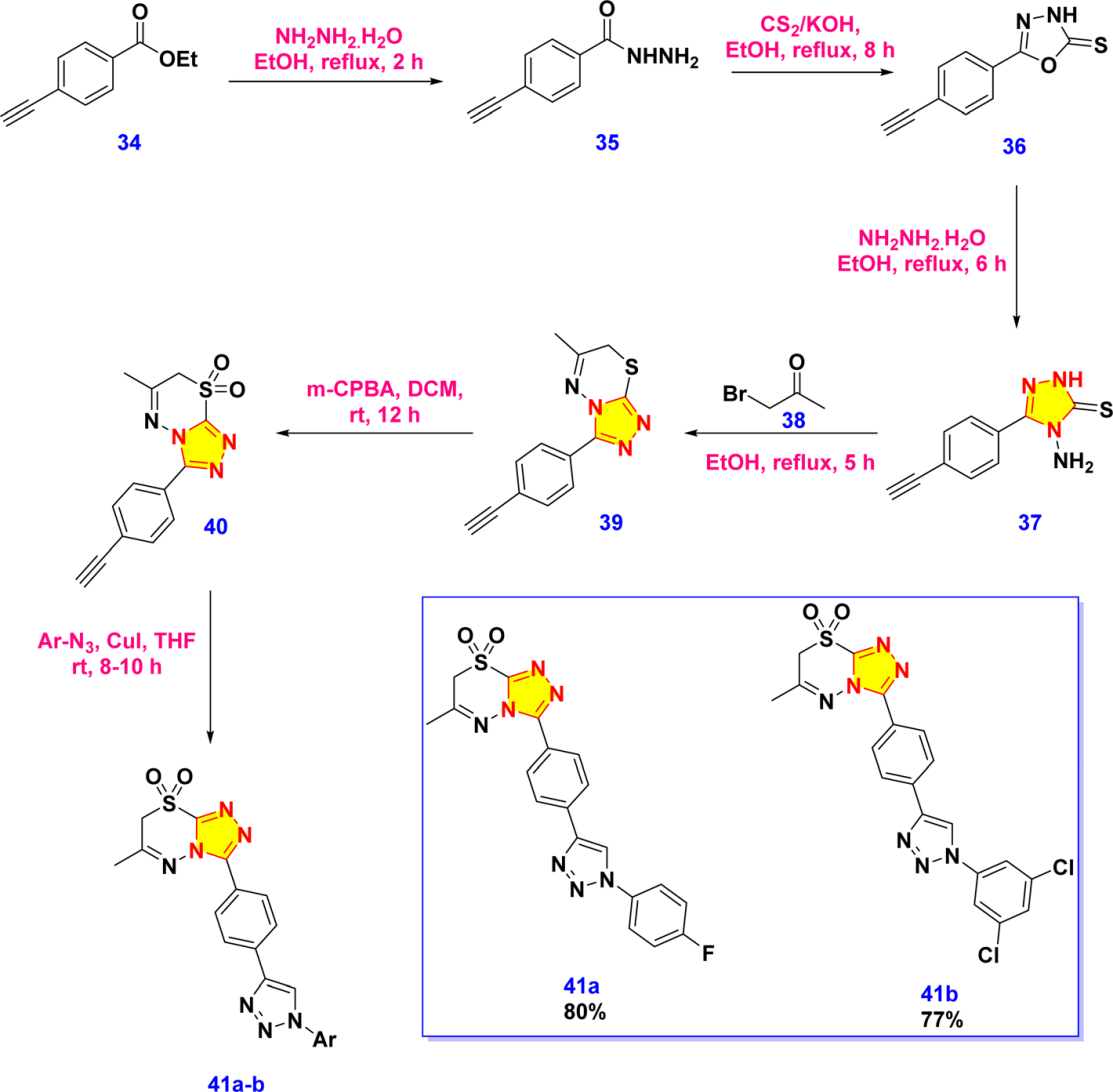
Synthesis of 1,2,3-triazoles linked with 1,2,4-triazoles 41a–b with EGFR inhibition.

1,2,3-Triazoles connected to 1,2,4-triazole through a spacer phenyl moiety have shown tremendous effects over EGFR kinases. Similarly, compounds 41a–b were produced from the conversion of ethyl 4-ethynylbenzoate 34 to its corresponding hydrazide 35 and cyclizing it to 1,3,4-oxadiazole-5-thione 36 and further transforming it into 1,2,4-triazole-amine 37 using hydrazine hydrate was achieved in easy three-step reactions. Further, bromoacetone 38 was used for the ring expansion fused with 1,2,4-triazole 39. Thio group was then oxidized using *m*-CPBA in dichloromethane and treated with different aryl azides for copper catalyzed-1,3-dipolar cycloaddition to obtain 1,2,3-triazole linked to 1,2,4-triazole through a phenyl spacer group 41a–b. Compound 41a showed excellent inhibition towards EGFR kinases with an IC_50_ value of 0.419 ± 0.05 µM and compound 41b with 0.312 ± 0.02 µM compared with Erlotinib. In contrast to the previous case, a phenyl spacer moiety flanked in between 1,2,3-triazole and 1,2,4-triazole also results in enhanced EGFR kinase inhibition. Furthermore, halogen substituted phenyl groups attached to the 1,2,3-triazole moieties offers good hinge interactions. Additionally, they had potent activity against MCF-7 cancer cells with IC_50_ values of 4.10 ± 0.71 and 3.86 ± 0.51 µM, respectively. Molecular docking studies revealed few major interactions of compound 41a including H-bonding with Cys773 and π–π interactions with Asp831, Ala835, Met857, Leu838, Tyr845 and Phe699 whereas 41b also showed similar interactions except replacing H-bonding with Asn818 instead of Cys773 (Scheme 5).^104^

**Scheme 6:**
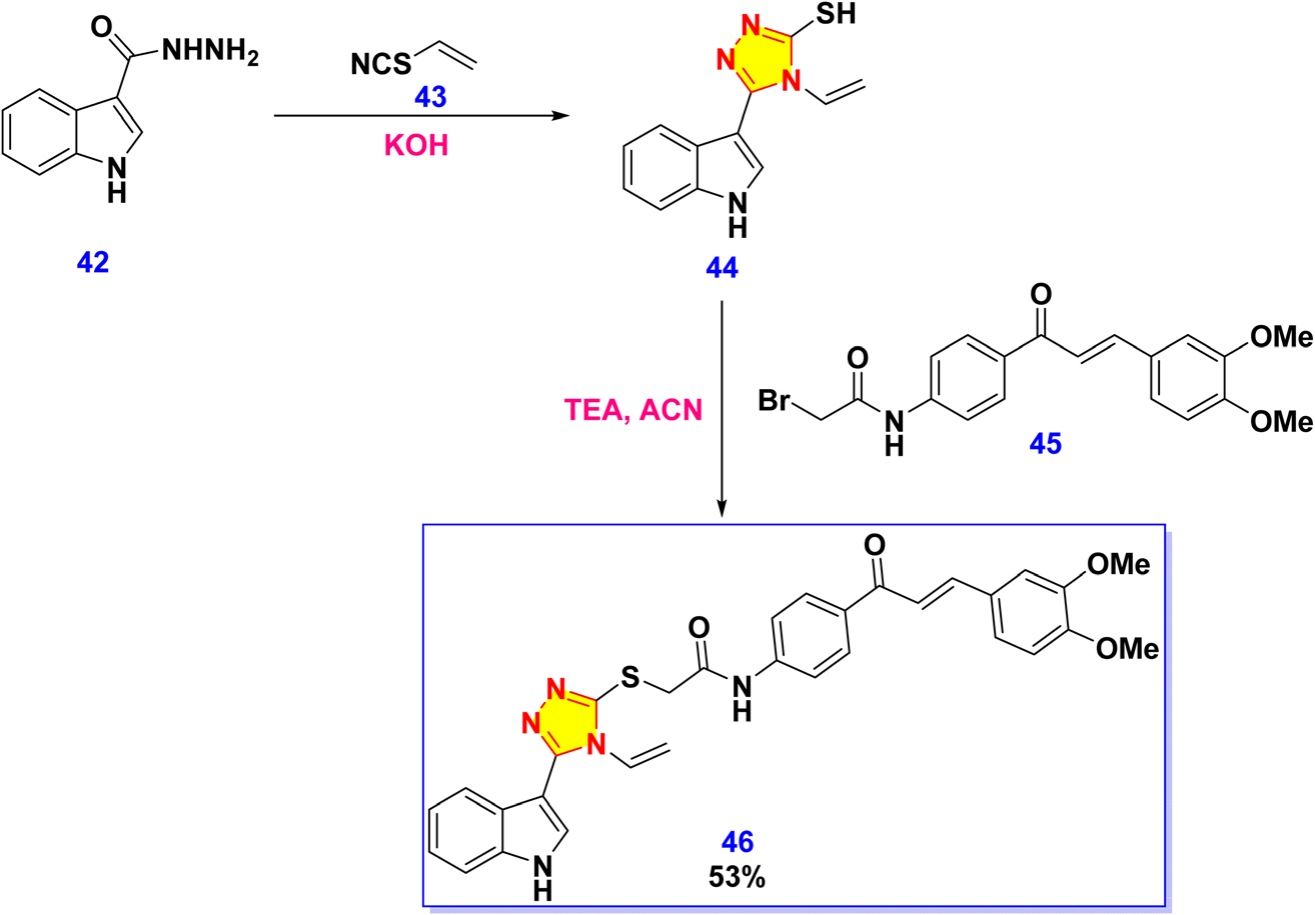
Synthesis of indole-linked 1,2,4-triazole chalcone 46.

Indole when linked with 1,2,4-triazole-extended chalcone moiety, show good activity against EGFR tyrosine kinase. Similarly, Mahmoud *et al.* synthesized compound 46 from indole-hydrazide 42 upon treating it with allyl thiocyanate 43 to give *N*-allyl 1,2,4-triazole 44 which was further treated with a chalcone-linked phenylacetamide 45 in the presence of triethylamine and acetonitrile. It showed its potency against EGFR kinase with an IC_50_ value of 0.052 ± 0.04 µM in reference with Erlotinib. However, its potency is not only limited to EGFR kinase inhibition, rather it is expanded towards different cancer cells such as MCF-7 and Panc-1 with IC_50_ values of 0.75 ± 0.08 and 1.30 ± 0.20 µM, respectively. Molecular docking studies revealed few of the major interactions which include H-bonding with Met769 and many electrostatic interactions with Asp776, Lys721, Thr766, Cys773 along with hydrophobic contacts with Ala719, Phe832, Leu820 (Scheme 6).^105^

**Scheme 7:**
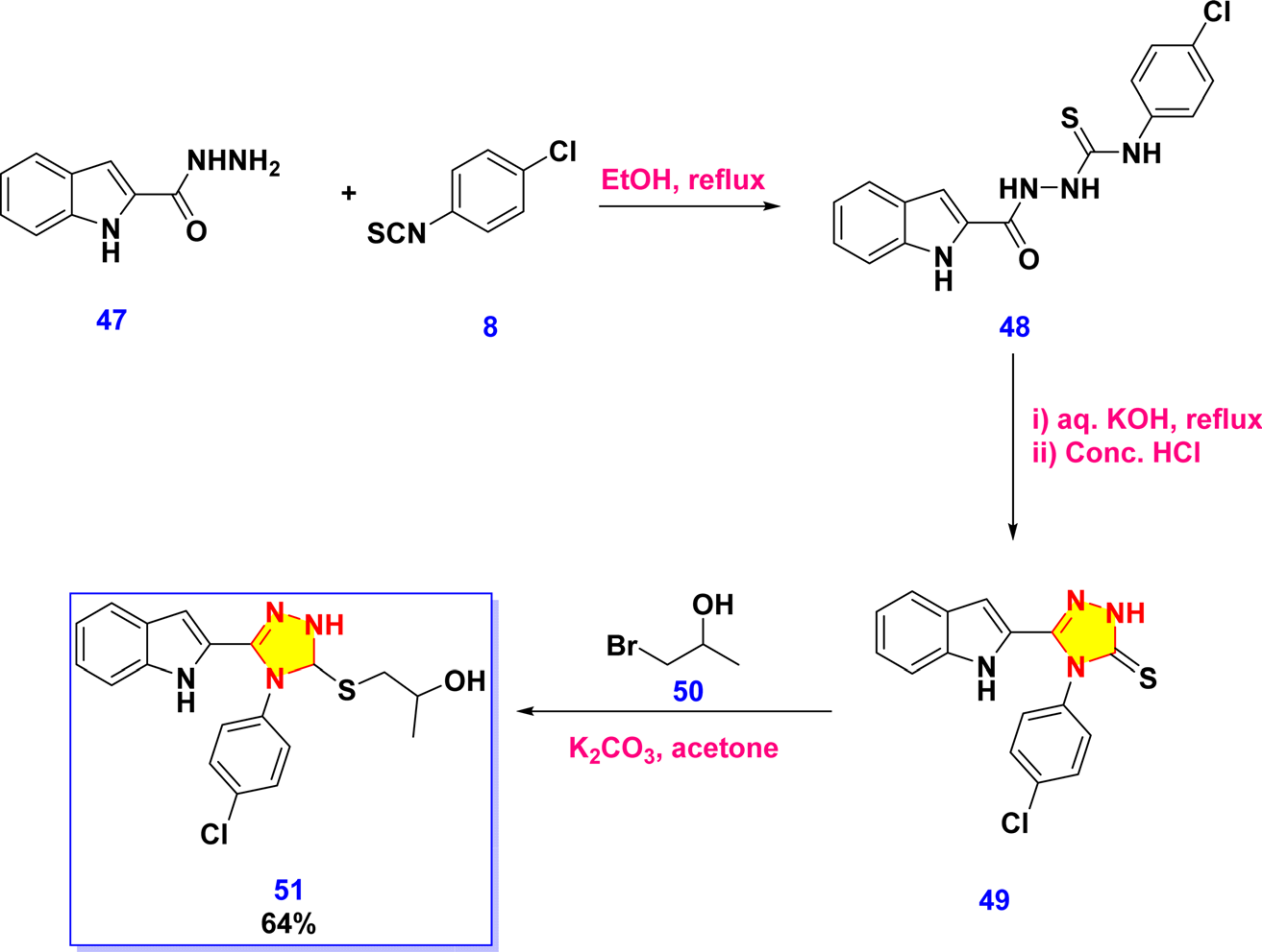
Synthesis of indole-based 1,2,4-triazole 51.

Youssef *et al.* produced indole-based 1,2,4-triazole 51 with good effect against EGFR kinase with IC_50_ value of 62.4 nM and against different cancer cells such as MCF-7 and HepG-2 with IC_50_ values of 1.07 and 0.32 µM when compared with Erlotinib. Its synthesis started from reaction indole-2-hydrazide 47 with 4-chlorophenylisothiocyanate 8 refluxed in ethanol to give thiosemicarbazide linker moiety 48 which was further refluxed in the presence of potassium hydroxide and neutralized using concentrated hydrochloric acid to give cyclized 1,2,4-triazole ring 49 and treated with 1-bromopropan-2-ol 50 in the presence of a base and acetone. Molecular docking analysis put-forth two major interactions such as one H-bond with Met793 and one arene–cation contact with Lys745 residue (Scheme 7).^106^Table 1 illustrates an overview of the so far discussed 1,2,4-triazole containing EGFR kinase inhibitors.

**Scheme 8:**
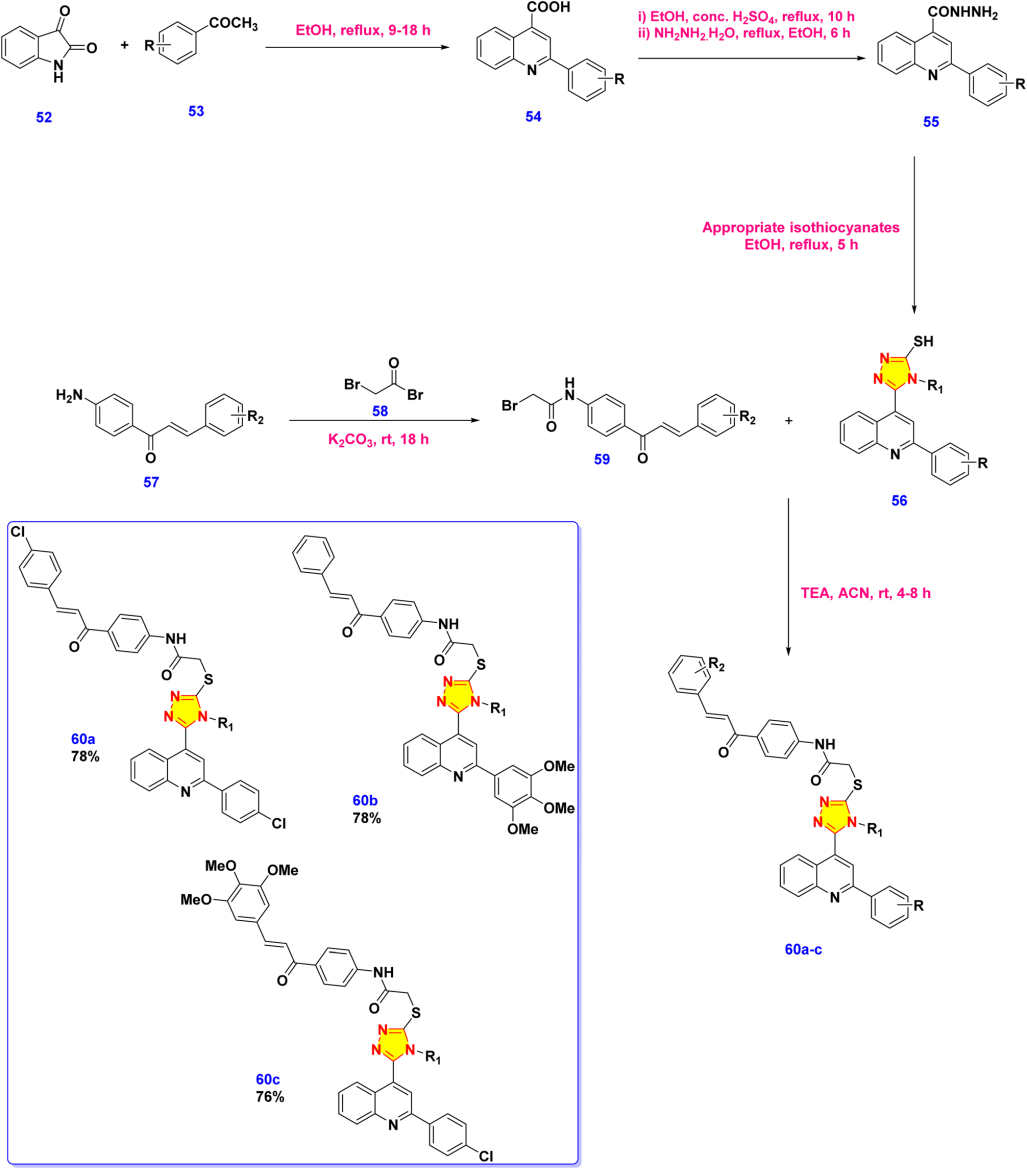
Synthesis of quinoline-based 1,2,4-triazole EGFR inhibitors 60a–c.

Isatin 52 undergoes ring expansion when treated with different acetophenones 53 to give quinolinic acid derivative 54 which is then esterified and corresponding hydrazide 55 is produced from hydrazine hydrate. The hydrazide moiety 55 undergoes cyclization in the presence of appropriate isocyanates to give 1,2,4-triazole ring 56 with thiol substituent. Meanwhile, different chalcone compounds 57 were treated with bromoacetyl bromide 59 to give another intermediate 59. These two intermediates 56 and 59 were combined in the presence of triethylamine and acetonitrile at room temperature to give target compounds 60a–c. All the three compounds 60a–c were found to be effective against EGFR kinases with IC_50_ values of 1.3 ± 1.2, 2.1 ± 0.4, 2.8 ± 0.8 µM. Furthermore, their activity against different cancer cells was also impressive with IC_50_ values of 3.6 ± 0.05, 3.3 ± 0.08, 2.9 ± 0.02, 3.5 ± 0.02 µM against A-549, MCF-7, Panc-1, HT-29 cancer cells, respectively. Similarly, compounds TE12 and TE13 also showed remarkable results when compared to standard drug Erlotinib. These target compounds showed variable molecular interactions with amino acid residues through docking studies which included H-bonding with Cys773, Thr766, Lys483 and Cys532 π–cation interaction with Lys721, π–sigma interaction with Val702, Leu820 and π–alkyl interaction with Met769 and Met742 (Scheme 8).^107^

**Scheme 9:**
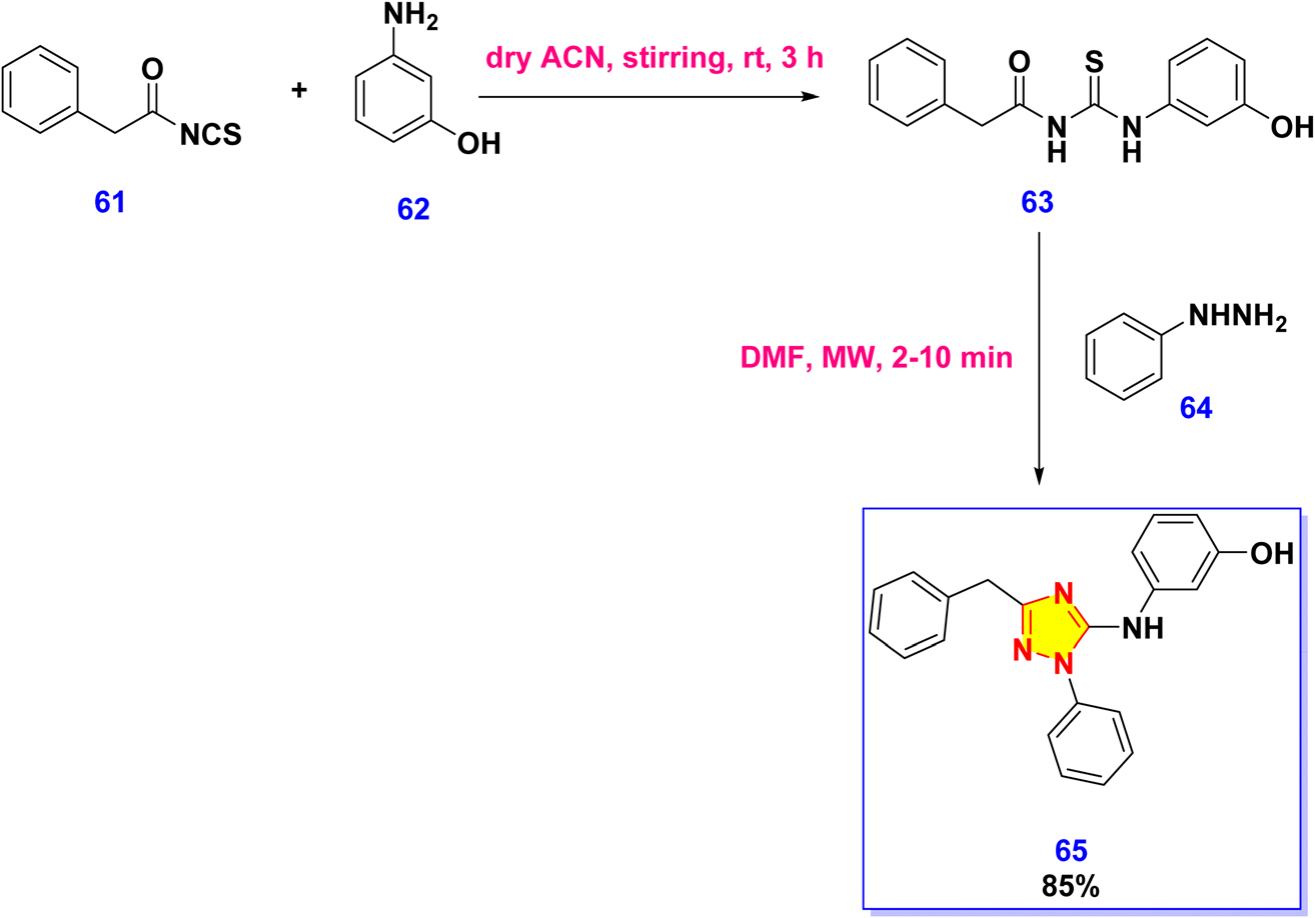
Synthesis of 3-aminophenol-derived 1,2,4-triazole EGFR kinase inhibitor 65.

Aminophenol derivatives have been interesting scaffolds of study with diverse biological effects. 2-Phenylacetyl isothiocyanate 61 when treated with 3-aminophenol 62 in the presence of dry acetonitrile and stirred at room temperature for about 3 h produced important intermediate 63 which upon treatment with phenylhydrazine 64 in the presence of DMF under microwave irradiation for 2–10 min gave target compound 65 in good yield. This compound 65 showed potential against two different EGFR kinases namely EGFR^WT^ and EGFR^T790M^ with IC_50_ values of 0.08 ± 0.05 and 0.09 ± 0.01 µM in comparison with Erlotinib. Additionally, its action was found to be promising across cancer cells including MCF-7 human breast cancer cells, A-549 lung cancer cells and HepG-2 hepatocellular cancer cells with IC_50_ values of 1.29 ± 0.03, 3.18 ± 0.03, and 5.47 ± 0.02 µM, respectively. These values were better than the standard reference drugs Erlotinib and Doxorubicin. Docking studies revealed several interactions responsible for enhanced EGFR kinase inhibition activity involving H-bonding with Lys721 and Met769 with arene–cation interaction with Val702 (Scheme 9).^108^

**Scheme 10:**
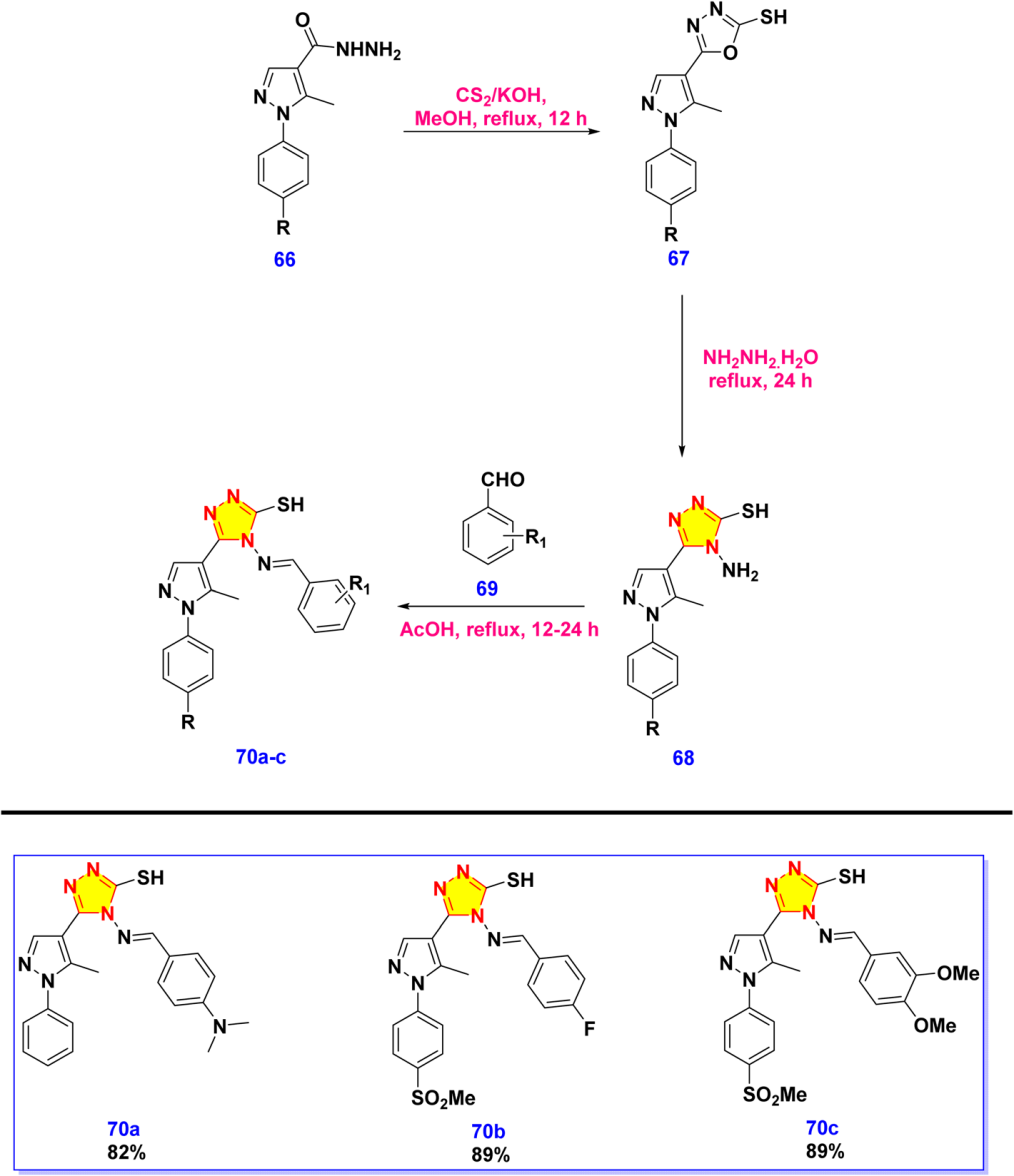
Synthesis of pyrazole-based 1,2,4-triazole derivatives 70a–c.

Pyrazole-1,2,4-triazole derivatives 70a–c were proved to be prominent in inhibiting EGFR kinases whose synthesis started from the reaction of pyrazolyl-hydrazide 66 and converting it into 1,3,4-oxadiazole-5-thiol 67 and further converting into 1,2,4-triazole-amine 68 using hydrazine hydrate. Later, various aromatic aldehydes 69 were treated with this 1,2,4-triazole-amine intermediate 68 and furnished target compounds 70a–c in high yields. Compounds 70a–c displayed promising action against EGFR^WT^ and EGFR^T790M^ with IC_50_ values of 0.423 ± 0.014, 0.121 ± 0.006, 0.216 ± 0.007 and 0.764 ± 0.023, 0.076 ± 0.002, 0.135 ± 0.004 µM, respectively. Moreover, their effect was more interesting against different cancer cells with IC_50_ values of 7.46 ± 0.32, 1.20 ± 0.05, 5.14 ± 0.22 µM against MCF-7 cancer cells, 2.93 ± 0.13, 5.14 ± 0.22, 9.23 ± 0.4 µM against HT-29 cancer cells, 3.78 ± 0.16, 4.89 ± 0.21, 2.38 ± 0.1 µM against A-549 cancer cells, respectively. These values were found to be better than the standard drugs used as references such as Erlotinib and Gefitinib. These compounds exhibited few major interactions with different amino acid residues such as H-bonding with Ser516, Arg499, His75. Alongside, hydrophobic contacts with Ala502, Val102, Leu338 and Val509. Alkyl interaction with Val735, Leu338, Ala513 and Val102. Noteworthy, compound 70c exhibited an unique amide–π stacked Asp501 interaction and π–sigma link with Ala502 (Scheme 10).^109^

**Scheme 11:**
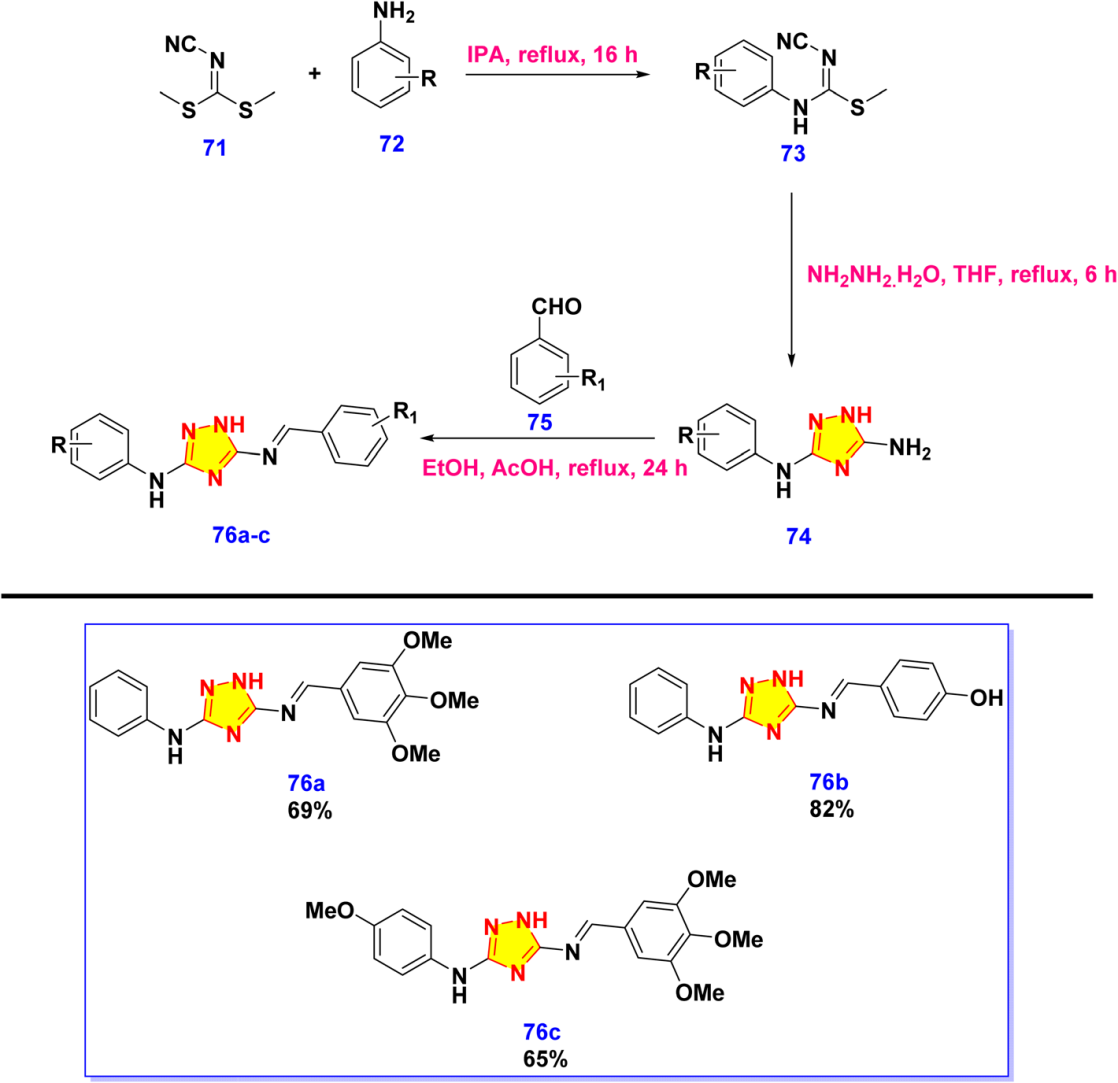
Synthesis of 1,2,4-triazole Schiff's bases 76a–c.

1,2,4-Triazole Schiff's bases 76a–c were generated using dimethyl cyanodithioimidocarbonate 71 reacted with various aromatic amines 72 in the presence of isopropanol for 16 h to give intermediate 73 which was treated with hydrazine hydrate in THF to form 1,2,4-triazole-5-amine 74. Finally, this amino group was made to react with different aryl aldehydes 75 to form Schiff's bases 76a–c. This straightforward three-step synthesis was well-facilitated with proper methods and reaction conditions since many years now. These target compounds 76a–c showcased its potential against EGFR kinase with IC_50_ values of 0.135 ± 0.017, 0.154 ± 0.021 and 0.225 ± 0.034 µM, respectively. These values were found to be very close to Erlotinib. Molecular docking studies showed major interactions including H-bonding with Cys751, Thr179, alkyl interaction with Ala719, Lys721, Leu820, Cys241, Leu248, Ala250, Ala316 and Leu254 (Scheme 11).^110^

**Scheme 12:**
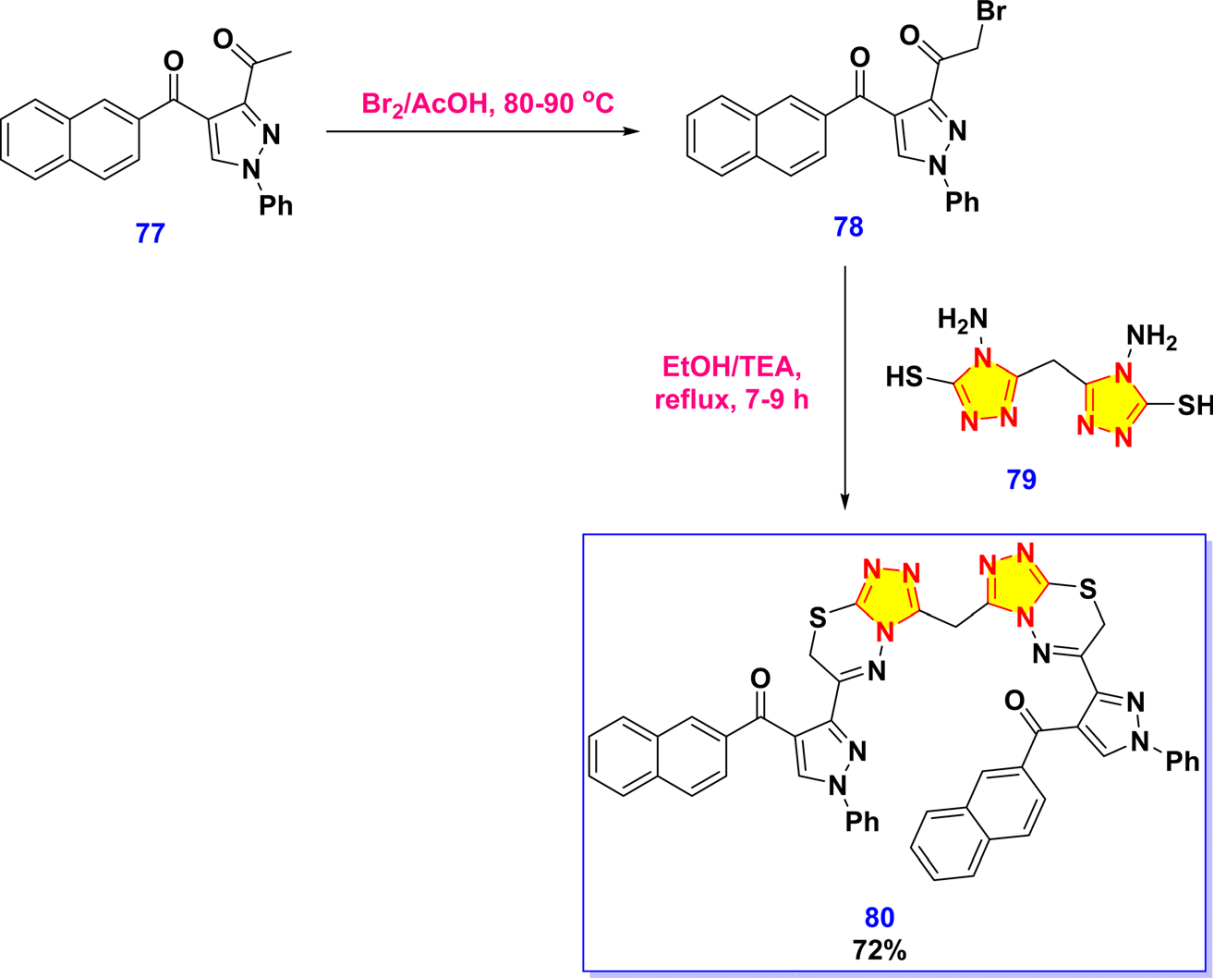
Synthesis of 2-naphthyl-derived pyrazole-1,2,4-triazole hybrid 80.

2-Naphthyl pyrazole-derived 1,2,4-triazole 80 is promising in its action towards combating the rapid division of cancerous cells. Compound 80 was prepared from brominating 1-(4-(2-naphthoyl)-1-phenyl-1*H*-pyrazol-3-yl)ethenone 77 in acetic acid at 80–90 °C to give 78 and later treated with bis-mercapto-1,2,4-triazoles 79 in the presence of ethanol in triethylamine refluxing for 7–9 h. It's excellent action against EGFR kinase was found with an IC_50_ value of 19.6 ± 0.64 µM much better than the standard drug Erlotinib. Moreover, its potentiality was further assessed by testing it against MCF-7 cancer cells and showed remarkable performance with IC_50_ value of 0.39 ± 0.1 µM even better than the standard drugs Erlotinib and Roscovitine. Docking interactions included H-bonding with Arg499 and arene–cation interaction with Val702 (Scheme 12).^111^

**Scheme 13:**
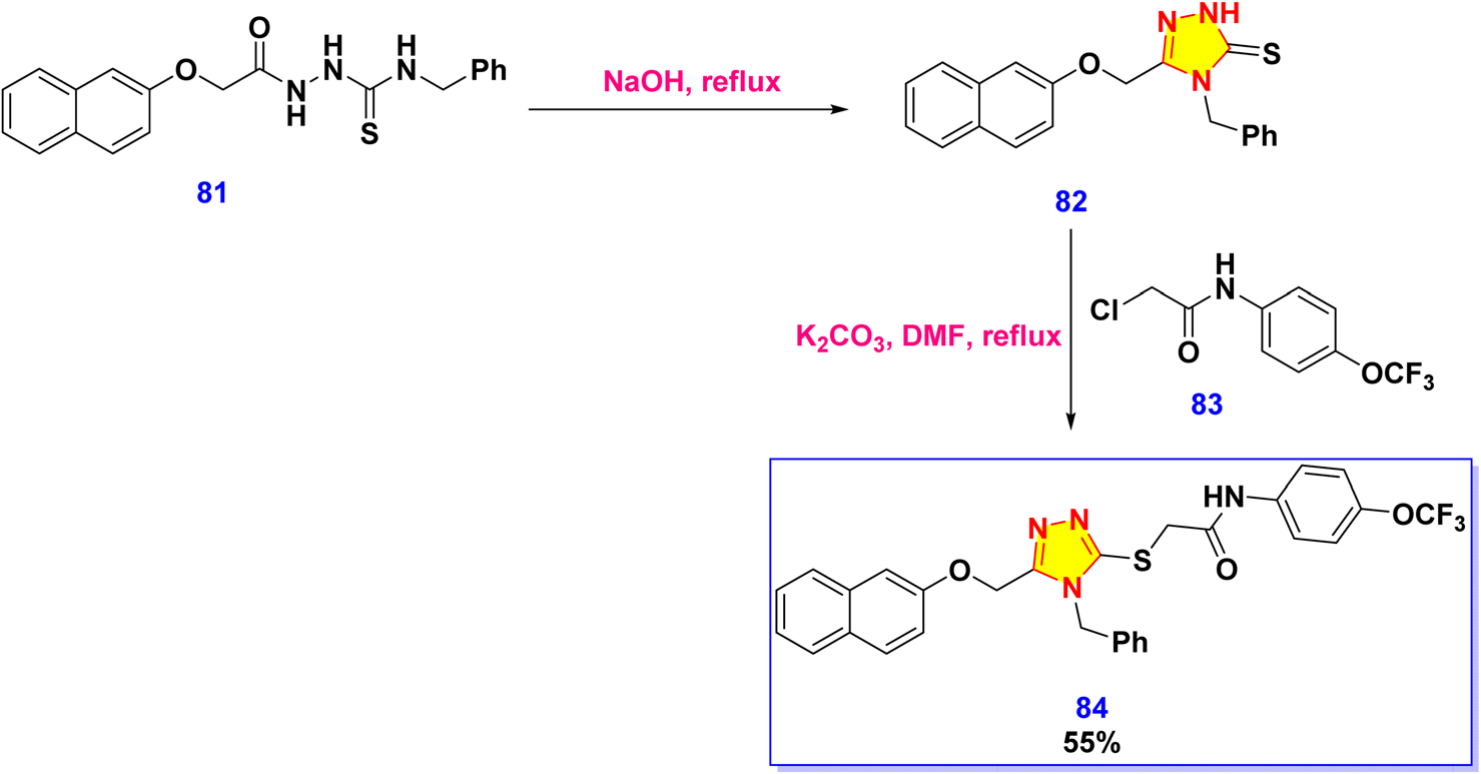
Synthesis of 2-napthoxy-derived phenylacetamide-based 1,2,4-triazole 84.

2-Naphthoxy-derived 1,2,4-triazoles linked to phenylacetamide 84 was prepared by cyclization of thiosemicarbazide moiety 81 using sodium hydroxide to give triazole 82 and combined with 2-chloro-*N*-(4-(trifluoromethoxy)phenyl)acetamide 83 in the presence of potassium carbonate and DMF. It inhibited EGFR kinase with IC_50_ value of 43.8 ± 1.3 nM. Additionally, it showed potency against PC-3 cancer cells with IC_50_ value of 3.18 ± 0.59 µM when compared to Gefitinib. Major interactions of compound 84 included H-bonding with Cys797 and van der Waals interaction with Ala763, Met766, Asn842, Tyr801, Asp800, Gly796, Cys797, Phe795, Met793, Thr790 (Scheme 13).^112^

**Scheme 14:**
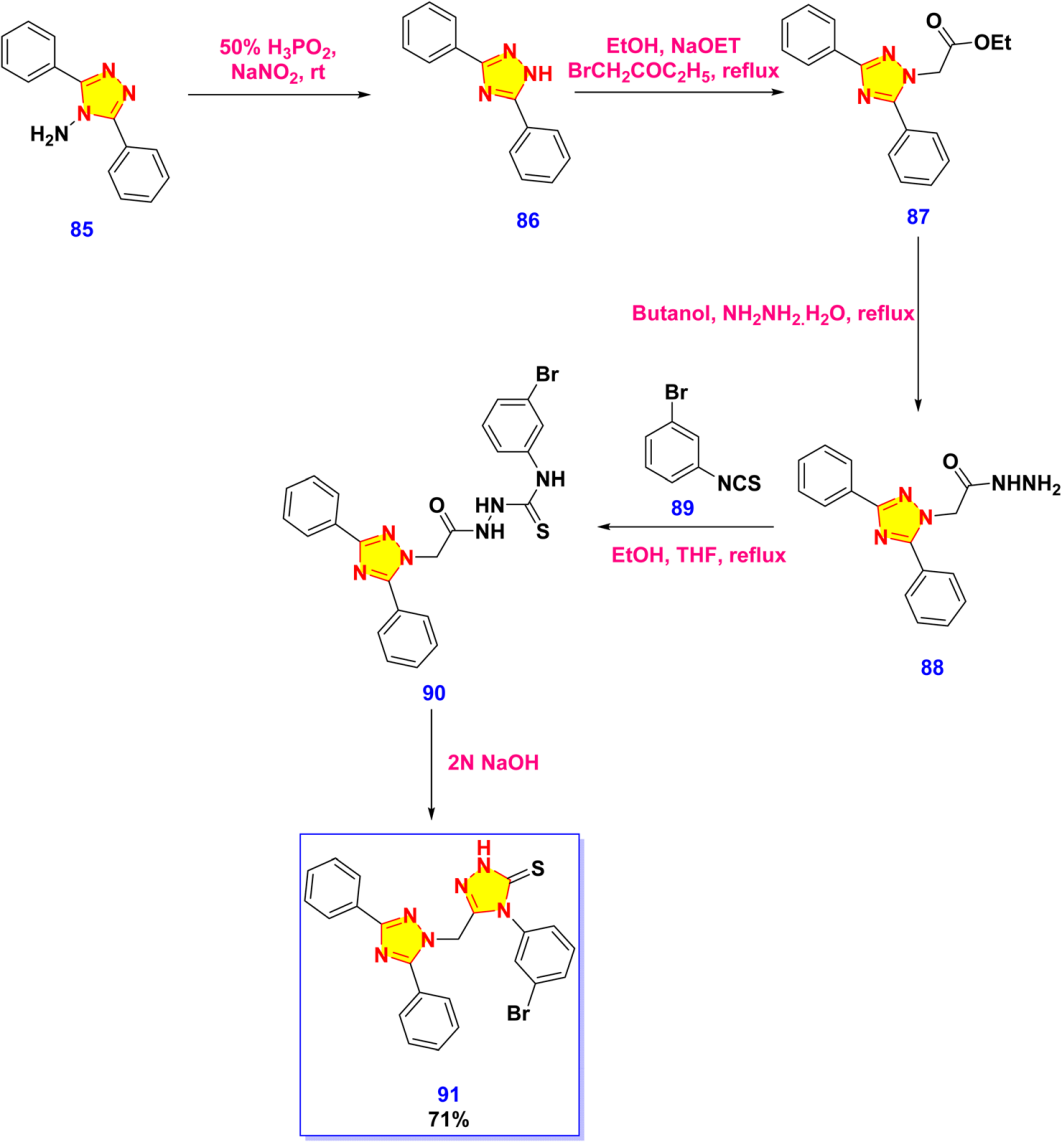
Synthesis of 3,5-diphenyl-1,2,4-triazole EGFR inhibitor 91.

1,2,4-Triazole when flanked with diphenyl groups enhanced the EGFR kinase inhibition activity. Moreover, when an additional triazole moiety is incorporated into this flanked system, the activity was much better. This was evident from their EGFR kinase inhibition activity results prior to which the compound 91 was synthesized from generating 1,2,4-triazole diphenyl 86 from the amine precursor 85 using H_3_PO_2_ in sodium nitrite and converting into an ester 87 and hydrazide 88 using hydrazine hydrate in butanol. Furthermore, treating 1-bromo-3-isothiocyanatobenzene 89 with the previously obtained intermediate 88, thiosemicarbazide moiety 90 was generated which was later cyclized using sodium hydroxide to for another 1,2,4-triazole moiety with methylene bridge 91. Its IC_50_ value against EGFR kinase was found to be 0.0082 mM which was better than the standard drug Gefitinib. Major interactions involved H-bond with Asn842 and Asp855 along with π–π stacking with Phe723 and Arg841 (Scheme 14).^113^Table 2 illustrates an overview of the so far discussed 1,2,4-triazole containing EGFR kinase inhibitors.

**Table 2:** Overview of 1,2,4-triazole-containing EGFR inhibitors

Compounds	Substituents	Major interactions	SAR observations
60a	4-Chlorophenyl rings attached to chalcone and quinoline linked to 1,2,4-triazole	H-bonding with Cys773 and Thr766. π–Sigma interaction with Val702 and Leu820. π–Alkyl interaction with Met769 and Met742	Electron-withdrawing two chloro groups attached to the phenyl ring activates the chalcone as well as electron-rich quinoline
60b	3,4,5-Trimethoxyphenyl ring attached to quinoline and a phenyl ring attached to chalcone moiety of 1,2,4-triazole hybrid	H-bonding with Lys483 and Cys532. π–Alkyl interaction with Leu514, Ile463 and Tyr538	Trimethoxy groups and electron-rich phenyl ring increased the activity
60c	4-Chlorophenyl group attached to quinoline and 3,4,5-trimethoxylphenyl ring attached to chalcone moiety of 1,2,4-triazole		Push-pull electronic effect tends to increase the EGFR kinase inhibition activity as seen in Sorafenib
65	Phenyl ring attached to *N*-of 1,2,4-triazole and benzyl group linked to C=N of triazole	H-bonding with Lys721 and Met769. Arene–cation interaction with Val702	Phenyl ring contributes extensively for the increased activity and benzyl group enhanced pharmacological profile
70a	Phenyl ring attached to pyrazole and 4-*N*,*N*-dimethylphenyl ring linked through imine of 1,2,4-triazole	H-bond with Ser516. Hydrophobic contacts with Ala502, Val702, Leu338 and Val509	Phenyl ring and *N*,*N*-dimethylphenyl ring activates triazole and pyrazole for increased activity
70b	4-Fluorophenyl ring attached to triazole through imine and methylsulfonylbenzene ring linked to pyrazole	H-bonding with Arg499 and His75. Alkyl interaction with Val735, Leu338, Ala513 and Val702	Phenyl ring and sulfonyl groups activate triazole and pyrazole for increased activity
70c	3,4-Dimethoxyphenyl ring attached to triazole through imine and methylsulfonylbenzene ring linked to pyrazole	H-bonding with Arg499. Amide–π stacking with Asp501. π–Sigma interaction with Ala502	Dimethoxy groups attached to phenyl ring and sulfonyl groups activate triazole and pyrazole for increased activity towards EGFR inhibition
76a	3,4,5-Trimethoxyphenyl ring connected to imine of 1,2,4-triazole and phenyl ring linked to triazole	H-bonding with Cys751. π–Alkyl interaction with Ala719, Lys721, Leu820	Electron-donating trimethoxy, hydroxy groups activated the triazole ring occupying the active site of EGFR kinase
76b	4-Hydroxyphenyl ring linked to imine and phenyl ring connected to triazole through –NH bond	H-bonding with Thr179. π–Alkyl interaction with Cys241, Leu248, Ala250, Ala316	
76c	3,4,5-Trimethoxyphenyl ring linked to imine and 4-anisyl ring attached to triazole through –NH bond	H-bonding with Thr719. π–Alkyl interaction with Lys254, Leu248, Ala250, Ala316	
80	2-Naphthoyl rings attached to pyrazolyl-traizole-fused thiazine	H-bonding with Arg499. Arene–cation interaction with Val702	Resonance stabilized naphthyl ring contributes greatly for the enhanced EGFR kinase inhibition
84	2-Napthoxy ring and benzyl group attached to triazole directly and trifluoromethoxy phenylacetamide moiety at one terminal end	H-bonding with Cys797. van der Waals interaction with Ala763, Met766, Asn842, Tyr801, Asp800, Gly796, Cys797, Phe795, Met793, Thr790	Trifluoromethoxy group increased the cytotoxicity
91	Two phenyl rings connected to triazole ring directly and a bromobenzene linked to triazole-thione	H-bond with Asn842 and Asp855. π–π stacking with Phe723 and Arg841	Electron-withdrawing bromo group enhanced EGFR kinase inhibition

### 2.2 VEGFR-2 kinase inhibitors

**Scheme 15:**
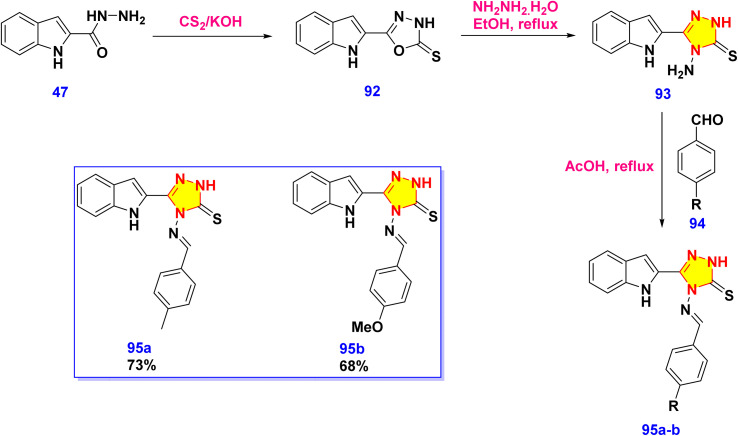
Synthesis of indolyl-1,2,4-triazole Schiff's bases 95a–b.

Indolyl-1,2,4-triazole hybrids 95a–b showed good potency against VEGFR-2 kinase and were produced by reacting 1*H*-indole-2-carbohydrazide 47 with CS_2_/KOH to give 1,3,4-oxadiazole ring 92 which was transformed into triazole amine 93 using hydrazine hydrate in ethanol. This intermediate 93 was then treated with different aldehydes 94 to form triazole-aldehyde linked Schiff's bases 95a–b. These Schiff bases 95a–b were prominent in their action against VEGFR-2 kinase with IC_50_ values of 0.034 ± 0.001 and 0.064 ± 0.002 µM, respectively. Moreover, they exhibited good activity against CAKI-1 renal cancer cells with IC_50_ values of 3.23 ± 0.15 and 1.63 ± 0.08 µM, respectively. These values were much better than the standard drug Sunitinib. Molecular docking results reflected few major interactions of this active compound including H-bonding with Phe1047 and π–alkyl interaction with Ala866, Val899, Val916, Phe918, Lys920, Phe921 (Scheme 15).^114^

**Scheme 16:**
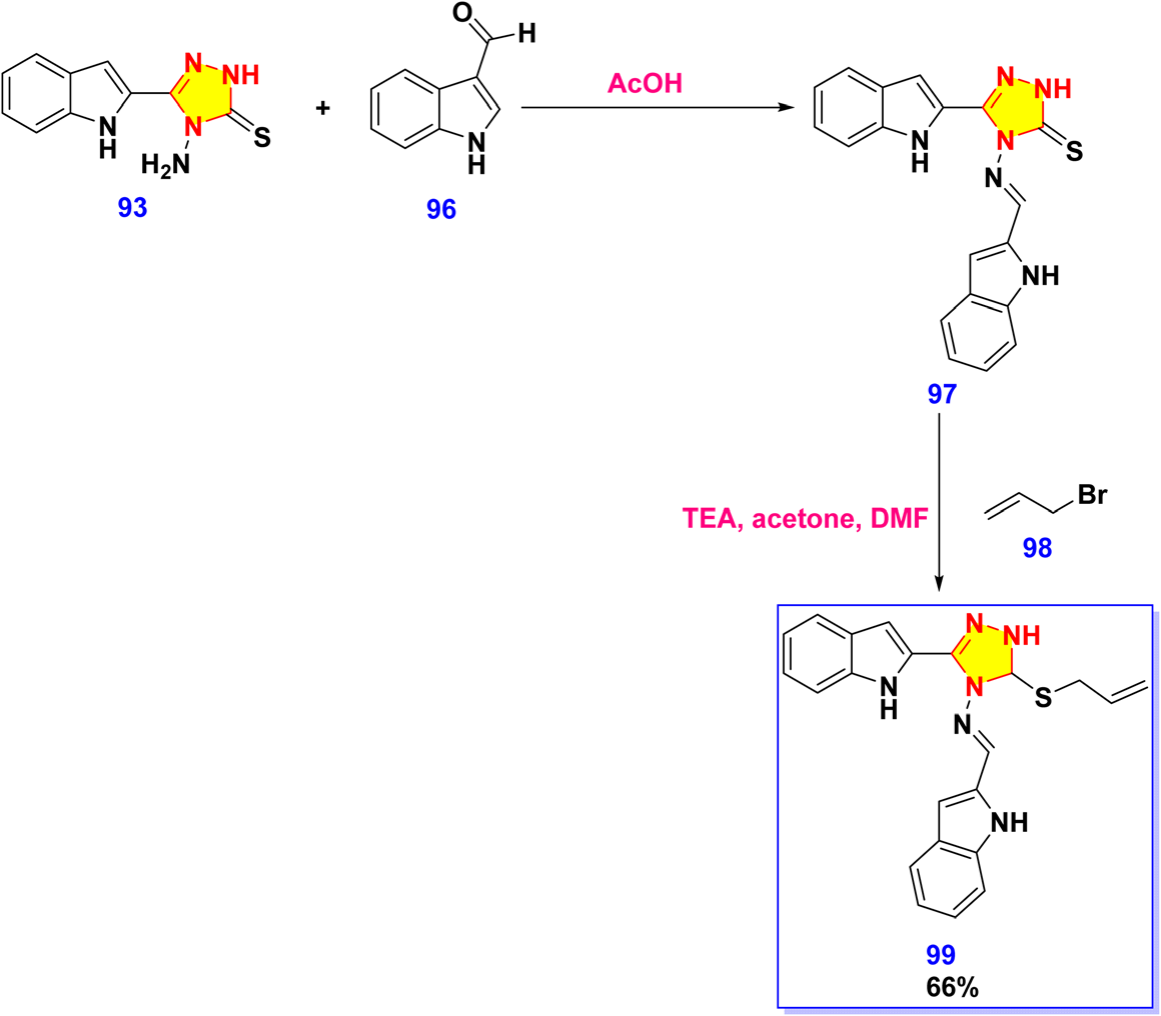
Synthesis of *S*-allylated indolyl-1,2,4-triazole analogue 99.

Alkylated indolyl-1,2,4-triazole derivatives were brilliant in their action against VEGFR-2 kinases. Especially, compound 99 was prepared from indole-triazole thione 93 and indole-3-carbaldehyde 96 in the presence of glacial acetic acid to form a *N*-alkylated Schiff's base 97 which was later treated with 3-bromoprop-1-ene 98 in triethylamine to form *S*-alkylated product 99 in moderate yield. This compound 99 was later investigated for its activity against VEGFR-2 kinase and different cancer cell lines for anticancer evaluation. As a result, it reflected better activity against VEGFR-2 kinase with IC_50_ value of 19.8 nM compared to Sunitinib. Also, it showed good action against MCF-7 with IC_50_ value of 1.18 ± 0.15 µM and HepG-2 cancer cells with IC_50_ value of 7.09 ± 0.67 µM, respectively. Docking studies revealed interactions such as H-bonding with Cys919. π–Cation interaction with His816, Arg1027. HB interaction with Asp1046 (Scheme 16).^115^

**Scheme 17:**
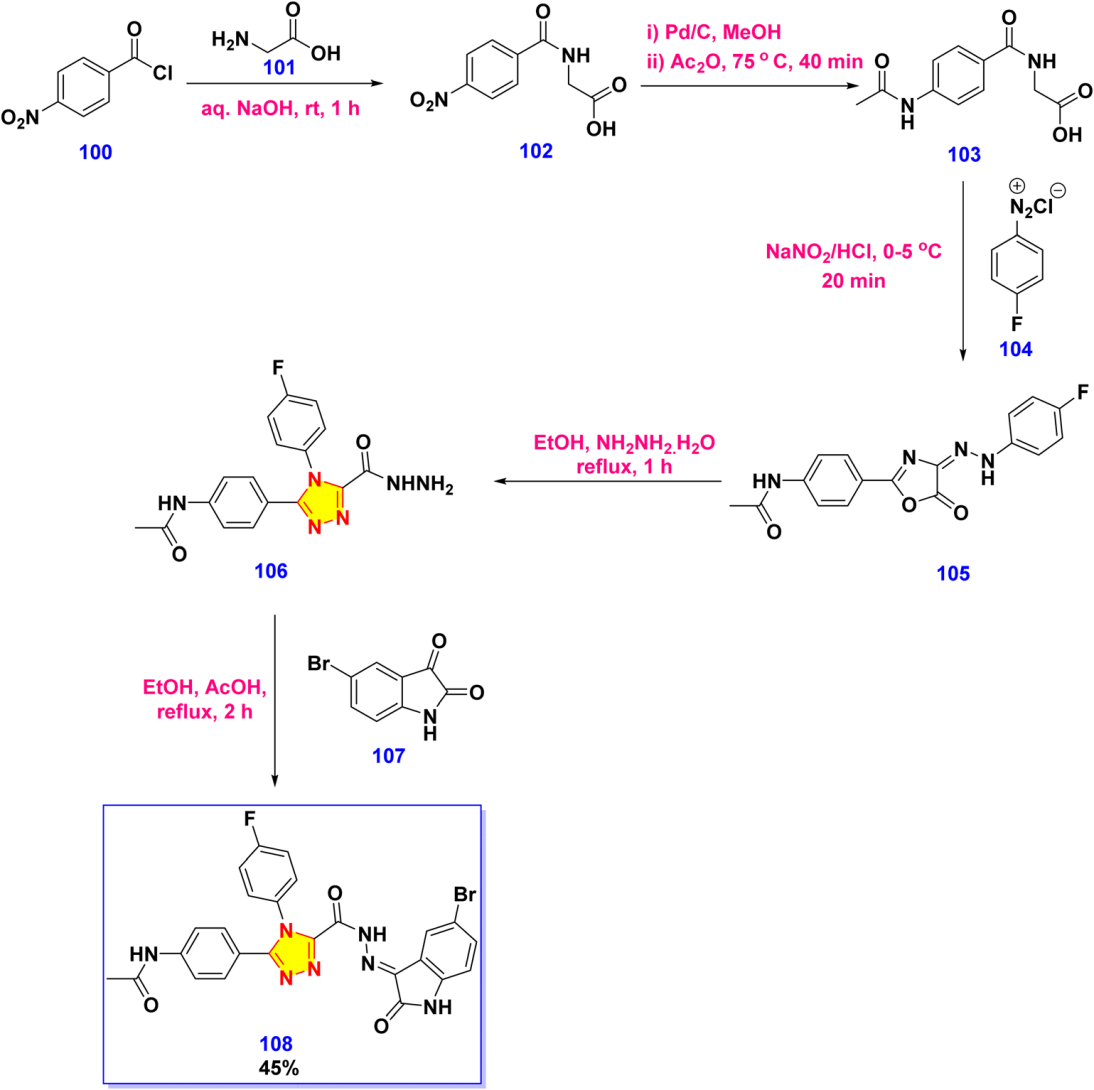
Synthesis of 1,2,4-triazole clubbed indolinone VEGFR-2 kinase inhibitor 108.

1,2,4-Triazole-clubbed indolinone 108 displayed promising performance against VEGFR-2, Panc-1 and HepG-2 cancer cells. This active compound 108 was synthesized from 4-nitrobenzoyl chloride 100 reacting with 2-aminoacteic acid 101 in the presence of NaOH to form a condensed product 102. The nitro group was then reduced to amine using Pd/C and the aminoacetic acid part is cyclized to oxazolone 103 which then made to form Schiff's base 105 with 4-fluorobenzene diazonium chloride 104 using sodium nitrite under freezing conditions. The oxazole 105 core part was then transformed into triazole using hydrazine hydrate in ethanol and refluxed for an hour to give an important pre-final compound 106 which was later treated with indolinone or bromo-substituted isatin 107 to furnish the final compound 108. This compound 108 was found to be effective against VEGFR-2 kinase with IC_50_ value of 16.3 ± 0.42 µM which was much superior than Sorafenib. Additionally, its action was excellent against Panc-1 and HepG-2 cancer cells with IC_50_ values of 1.16 ± 0.02 and 0.73 ± 0.0 µM, respectively. These two values were very close to Doxorubicin. Docking studies displayed major interactions involving four H-bonds with Asp1046, Lys868, Glu885 and Arg1027. Halogen bonding with Leu840. π–alkyl interactions with Val848, Ala866, Leu840, Leu889, Val898, Val899, Cys919 (Scheme 17).^116^

**Scheme 18:**
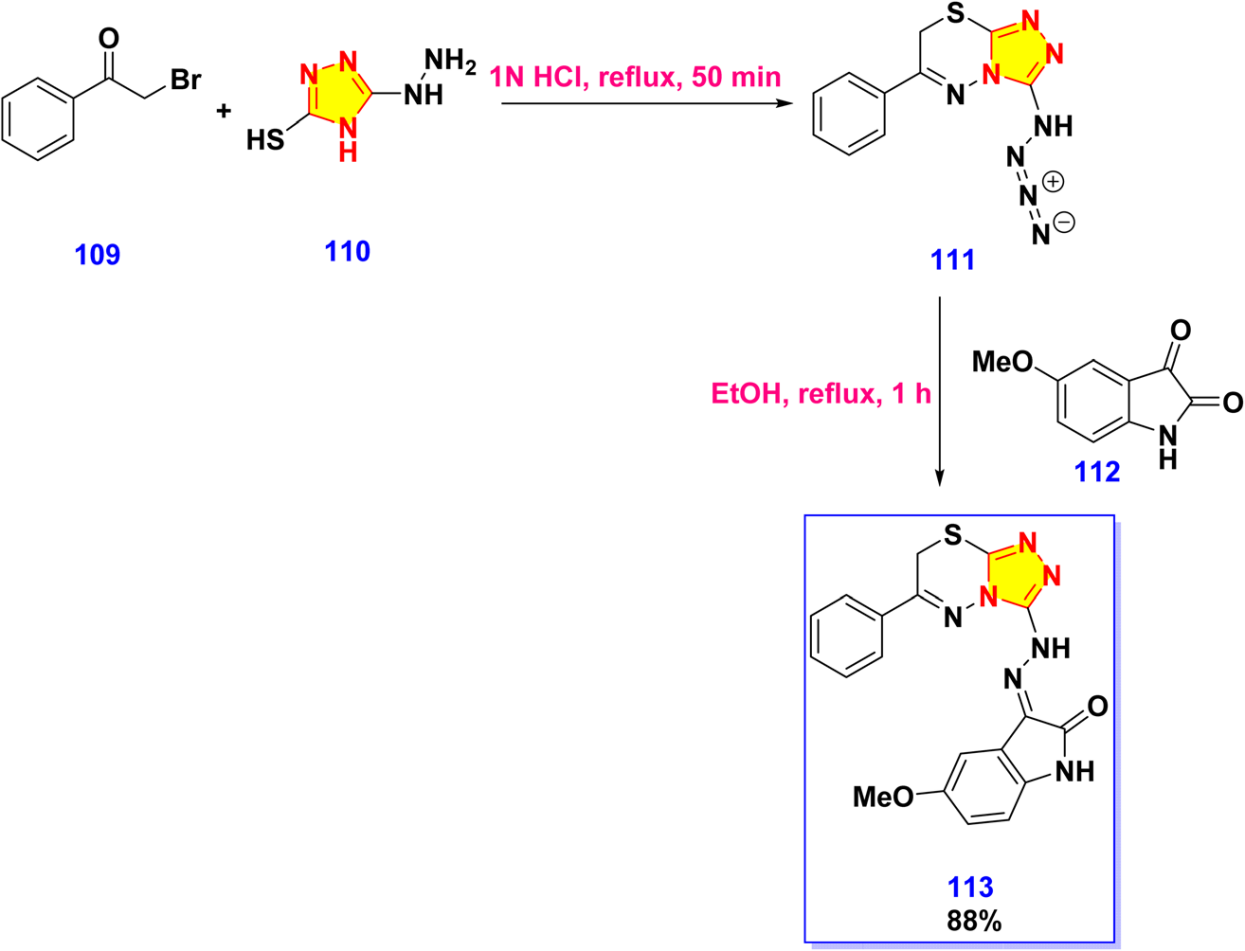
Synthesis of thiadiazine-triazole linked isatin hybrid 113.

3-(Triazolo-thiadiazin-3-yl)indolin-2-one analogue 113 was found to be excellent in its action against VEGFR-2 kinase with IC_50_ value of 435 nM compared to Sunitinib. Erstwhile, this compound 113 was a good angiogenesis inhibitor with its potent action against VEGFR-2 in combating the formation of tumour hijacked blood vessels and cells. This active compound 113 was prepared from phenacyl bromide 109 and 1,2,4-triazole amine 110 which led to the formation of thiadiazine-triazole fused triazonoium salt 111 which was later treated with 5-methoxy isatin 112 to give Schiff's base compound 113. Major interactions included two H-bonds with Glu917 and Cys919. Alongside, π–cation contacts with Lys838 and Leu840 (Scheme 18).^117^

**Scheme 19:**
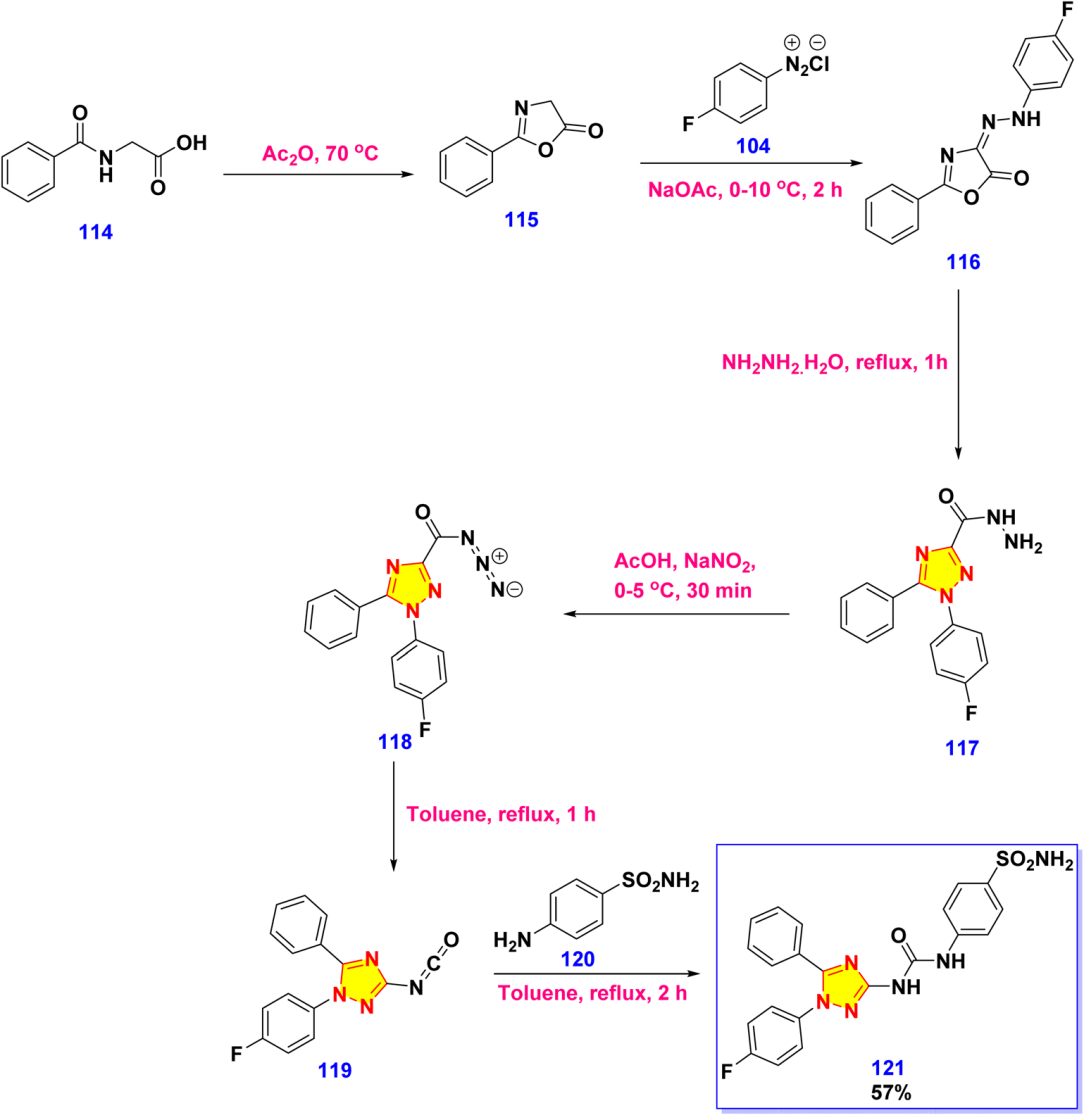
Synthesis of 1,2,4-triazole-urea hybrid 121.

1,2,4-Triazole urea 121 was seen to possess excellent activity against VEGFR-2 which was produced from 2-benzamidoacetic acid 114 and cyclized to oxazolone 115 using acetic anhydride. Further, it was combined with 4-fluorobenzenediazonium chloride 104 in sodium acetate to generate an aromatic imine 116 which was further treated with hydrazine hydrate to transform oxazole part into triazole 117. The hydrazide part was then converted into triazonium compound 118 and treated with toluene to give isocyanate 119 which was formed into urea 121 using 4-aminobenzenesulfonamide 120 in toluene under reflux conditions for 2 h. Compound 121 possessed good activity against VEGFR-2 kinase with IC_50_ value of 26.3 ± 0.4 µM, much better than Sunitinib. Furthermore, the evaluation against different cancer cell lines provided interesting results which included IC_50_ values of 0.66 ± 0.04 µM against MCF-7, 4.51 ± 0.2 µM against T47D, 25.58 ± 0.97 µM against MCF-10A and selectivity index of 38.76. These values were found to be better than Staurosporine. There were major interactions observed *via* docking studies which involved two H-bonds with Asp1046 and Cys919 along with π–π stacking with Phe1047. Hydrophobic contacts with Cys919, Val848, Leu840, Glu917, Val916 and Ala866 (Scheme 19).^118^

**Scheme 20:**
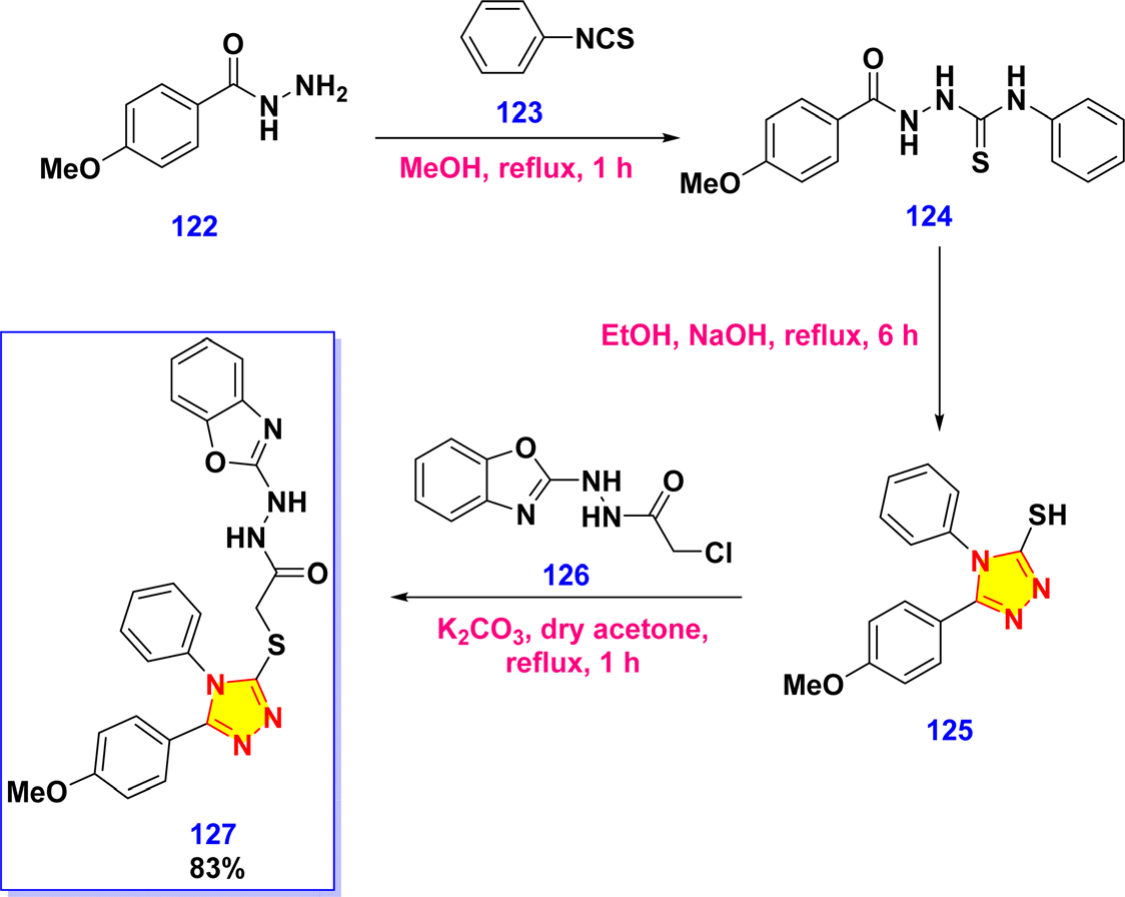
Synthesis of benzoxazole-derived 1,2,4-triazole VEGFR-2 kinase inhibitor 127.

Benzo[*d*]oxazole derivatives alone have been promising in their action against several kinases. Additionally, when they are combined with 1,2,4-triazole, their activity showed tremendous transition and effectiveness was more promising. Therefore, compound 127 was generated from 4-anisylbenzohydrazide 122 and treating it with phenyl isothiocyanate 123 in the presence of methanol to give condensed product 124 which was then treated with sodium hydroxide for the cyclization of thiosemicarbazide part to 1,2,4-triazole 125. This key intermediate compound 125 was further combined with benzoxazole derivative 126 in the presence of potassium carbonate and dry acetone in standard reflux conditions to accomplish target compound 127 in good yield. This compound 127 displayed its potency against VEGFR-2 kinase with IC_50_ value of 0.15 ± 0.01 µM whereas their effectiveness was further extended towards A-549 cancer cells with IC_50_ value of 13.12 ± 0.59 µM and HepG-2 cancer cells with 22.62 ± 1.12 µM, all compared with Sorafenib. Major interactions included two H-bonds with Asp1046 and Cys919. Hydrophobic interactions with Phe918, Cys919, Ile1044, Glu885, Val848, Val914 (Scheme 20).^119^

**Scheme 21:**
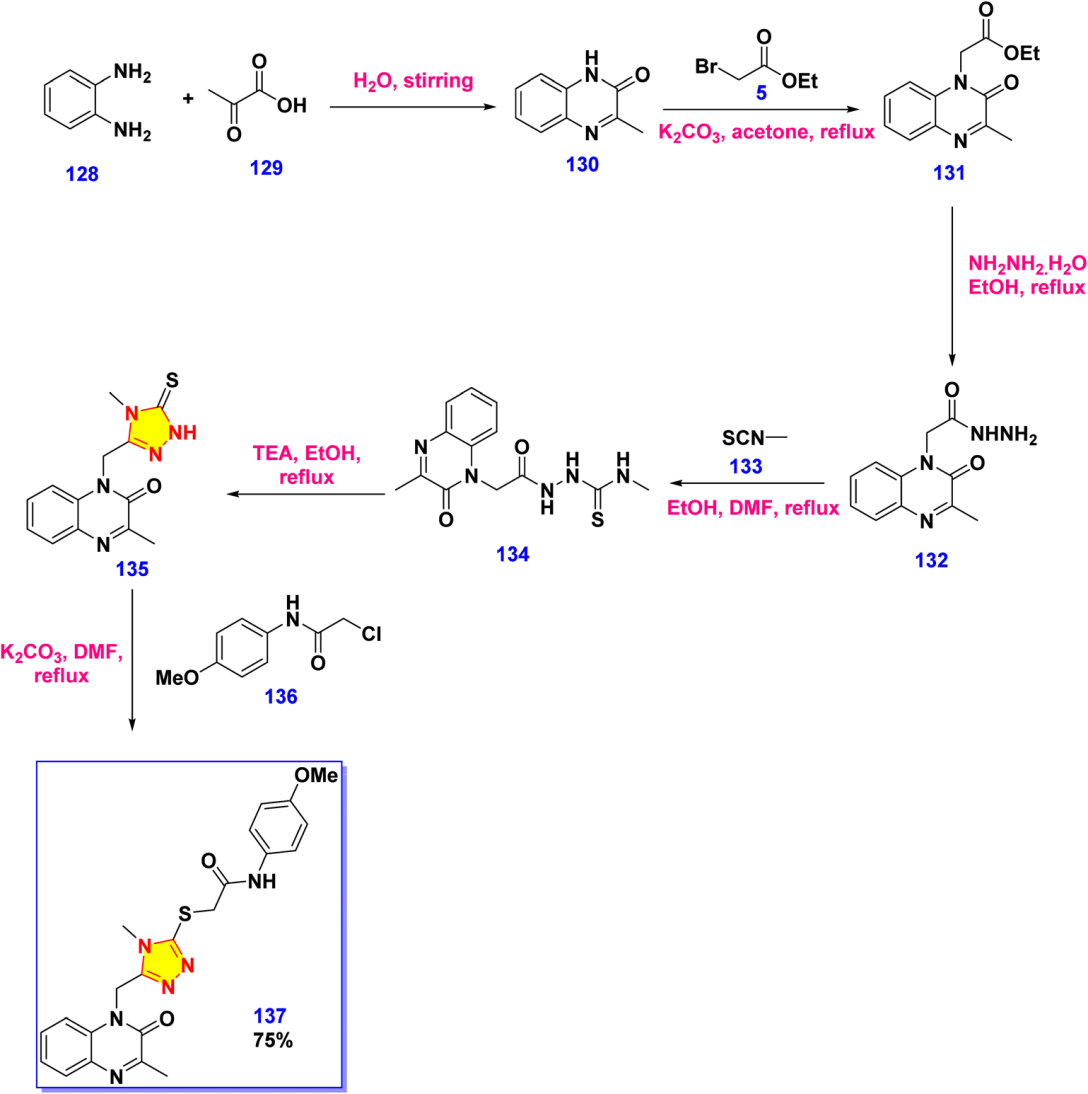
Synthesis of quinoxaline-derived 1,2,4-triazole hybrid 137.

Qunioxaline-derived 1,2,4-triazole 137 have dragged their attention due to their versatility in the field of bioorganic chemistry. Its synthesis started from the reaction between *o*-phenylenediamine 128 and 2-oxopropanoic acid 129 in water to give quinoxaline-core molecule 130 which was reacted with ethyl-2-bromoacetate 5 for the *N*-alkylation of quinoxaline 130. The ester part 131 was converted to hydrazide 132 using normal reaction with hydrazine hydrate and its treatment with methylisothiocyanate 133 to generate thiosemicarbazide derivative 134 which was then cyclized to 1,2,4-triazole-5-thione 135 using triethylamine in ethanol. Lastly, the pre-final compound 135 was combined with 2-chloro-*N*-(4-methoxyphenyl)acetamide 136 to give final compound 137 in good yield. This compound 137 showed its effectiveness against VEGFR-2 kinase with IC_50_ value of 0.037 ± 0.002 µM. Additionally, this compound 137 also had its effect against MCF-7 and MCF-10A with IC_50_ values of 1.6 ± 0.03 µM, 28.3 ± 0.6 µM in comparison with Sorafenib and Staurosporine. Few of the major interactions included three H-bonds with Glu885, Cys919 and Asp1046. π–Alkyl interaction with Ala866, Val916, Val899, Lys868, His1026 and Leu1019 (Scheme 21).^120^

**Scheme 22:**
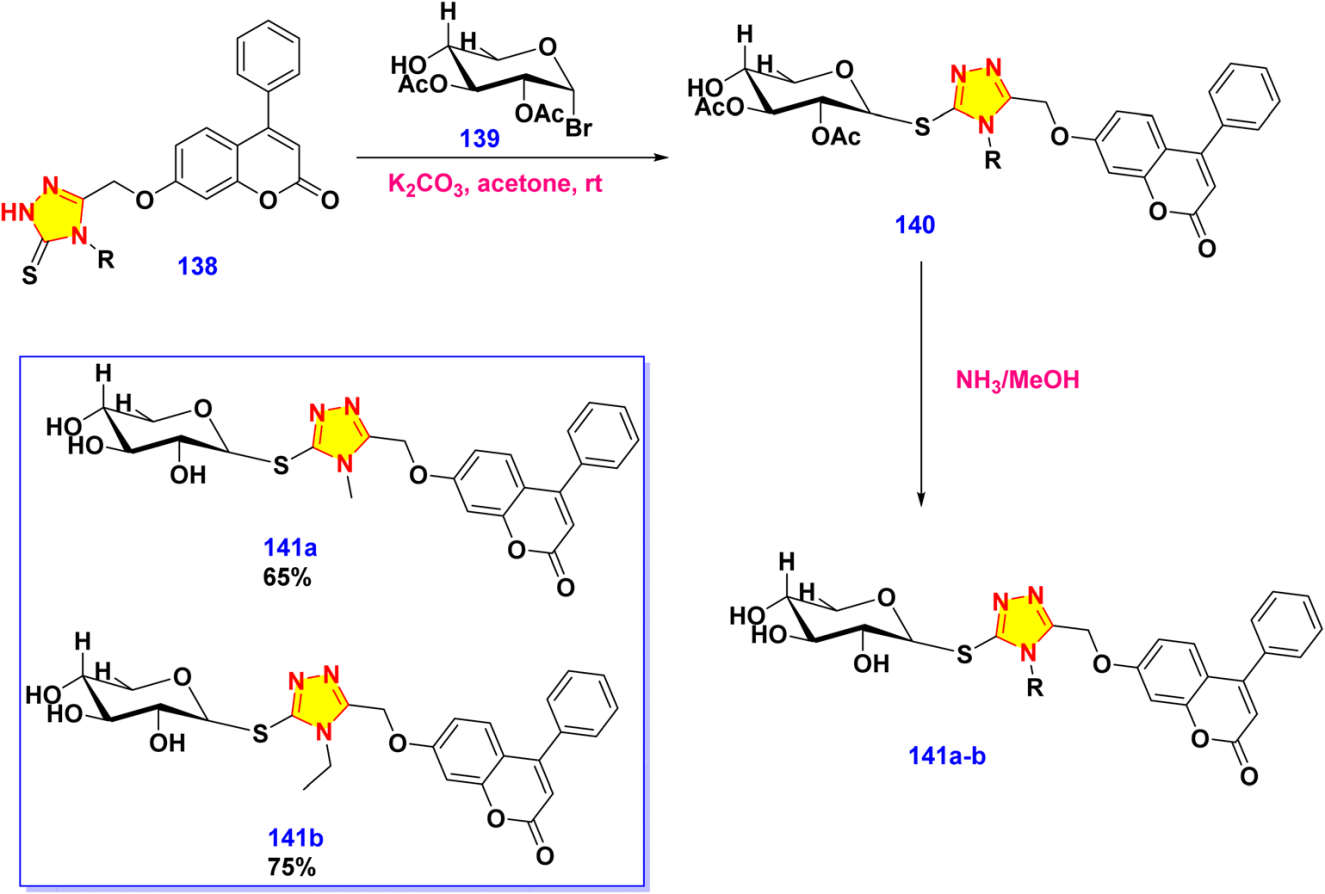
Synthesis of glycoside-coumarin-based 1,2,4-triazole analogues 141a–b.

Glycosides also have proven to be active compounds as anticancer agents in the field of medicinal chemistry. When they are clubbed with heterocyclic motifs, they tend to possess or enhance their biological performance. Likewise, glycosides-containing 1,2,4-triazoles coumarins 141a–b were constructed *via* two-step reaction involving condensation of 1,2,4-triazole coumarin 138 with a glycoside bromide 139 and later deprotecting the acetyl groups using ammonia in methanol to achieve the final compounds 141a–b in moderate to good yields. Altogether, when their anticancer activity was evaluated, it was known that these compounds 141a–b possessed high potential against VEGFR-2 kinases with IC_50_ values of 0.93 ± 0.42 and 0.79 ± 0.14 µM, respectively. Molecular docking studies displayed interactions such as H-bond with Cys919. Arene–cation interaction with Lys868, Leu889 and Cys1045 (Scheme 22).^121^Table 3 illustrates an overview of the so far discussed 1,2,4-triazole containing VEGFR-2 kinase inhibitors.

**Table 3:** Overview of 1,2,4-triazole-containing VEGFR-2 inhibitors

Compounds	Substituents	Major interactions	SAR observations
95a	4-Tolyl ring attached to 1,2,4-triazole through imine bond	H-bonding with Phe1047. π–Alkyl interaction with Ala866, Val899, Val916, Phe918, Lys920, Phe921	Electron donating methyl and methoxy groups increased cytotoxicity with anticancer effect
95b	4-Anisyl ring attached to 1,2,4-triazole through imine bond	H-bonding with Phe1047. π–Alkyl interaction with Val848, Cys1045, Ala866, Val916 and Phe921	
99	Indolyl-groups attached to triazole hybrid	H-bonding with Cys919. π–Cation interaction with His816, Arg1027. HB interaction with Asp1046	The *S*-allyl group occupies the hinger region of VEGFR-2 kinase responsible for enhanced inhibition
108	4-Fluorophenyl ring linked to triazole with direct phenylacetamide linkage	Four H-bonds with Asp1046, Lys868, Glu885 and Arg1027. Halogen bonding with Leu840. π–Alkyl interaction with Val848, Ala866, Leu840, Leu889, Val898, Val899, Cys919	Fluro group activated the phenyl ring which triggered the resonance stabilized indole rings flanked triazole
113	Phenyl ring attached thiadiazine-fused triazole ring	Two H-bonds with Glu917 and Cys919. π–Cation contact with Lys838 and Leu840	Electron rich phenyl ring system contributed immensely for VEGFR-2 kinase inhibition as it occupies the terminal site of binding pocket
121	Phenyl and 4-fluorophenyl ring attached to triazole-urea sulfonamide hybrid	Two H-bonds with Asp1046 and Cys919. π–π stacking with Phe1047. Hydrophobic contacts with Cys919, Val848, Leu840, Glu917, Val916 and Ala866	Fluro group activated the phenyl ring which triggered the resonance stabilized benzene sulfonamide moiety for increasing inhibition rate
127	Phenyl and 4-anisyl rings connected to triazole hybrid	Two H-bonds with Asp1046 and Cys919. Hydrophobic interactions with Phe918, Cys919, Ile1044, Glu885, Val848, Val914	Methoxy groups and electron rich benzene rings stabilized triazole moiety by occupying the active site of VEGFR-2 kinase
137	4-Anisyl group attached through acetamide linkage to triazole hybrid	Three H-bonds with Glu885, Cys919 and Asp1046. Alkyl interaction with Ala866, Val916, Val899, Lys868, His1026 and Leu1019	Electron-donating methoxy group contributed electrons and stabilized triazole ring for increased cytotoxicity
141a–b	Glycoside-linked triazole hybrids	H-bond with Cys919. Arene–cation interaction with Lys868, Leu889 and Cys1045	Methyl and ethyl groups attached to nitrogen of triazole increased pharmacological activity

### 2.3 Carbonic anhydrase inhibitors

**Scheme 23:**
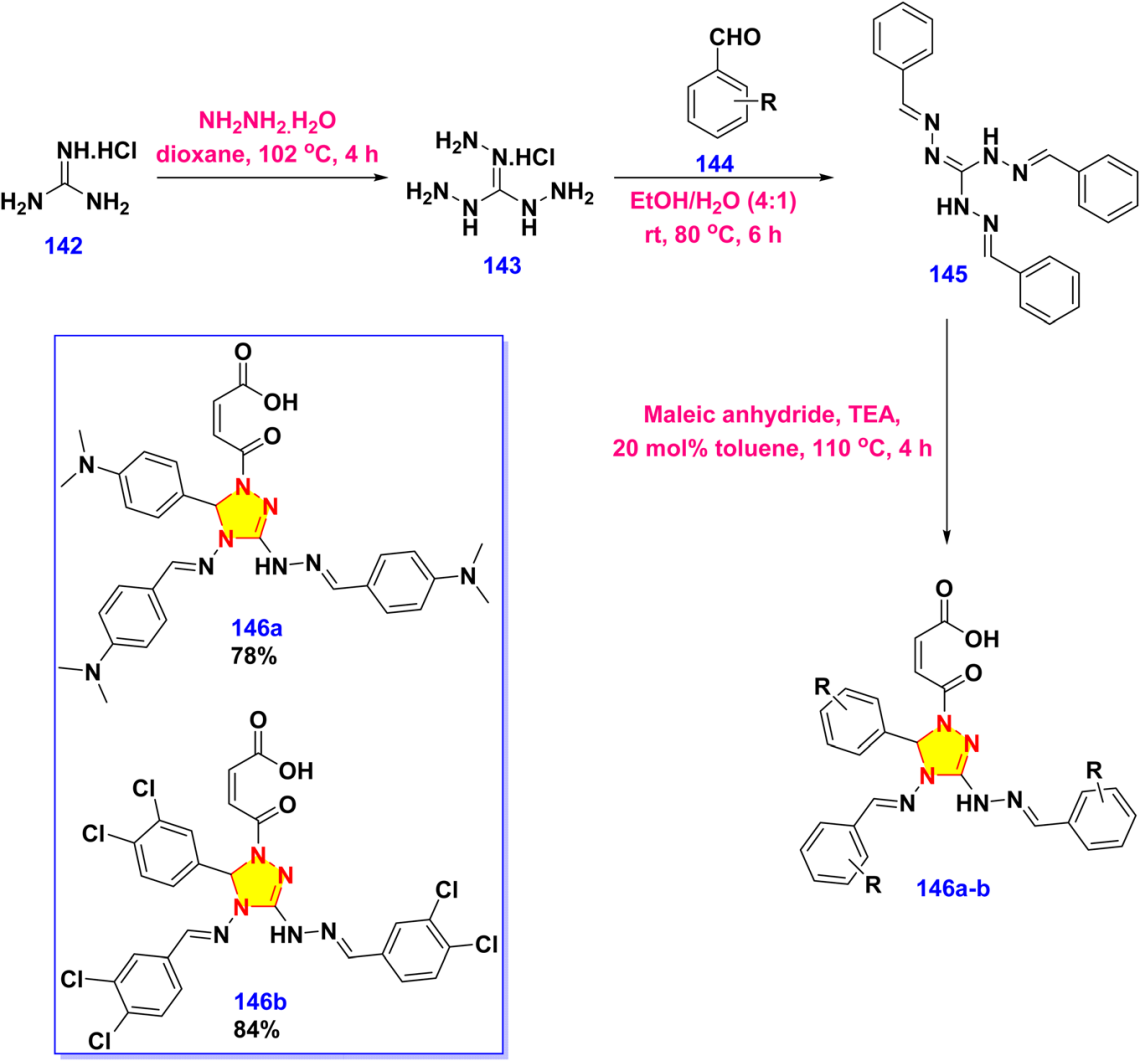
Synthesis of tris-Schiff's bases-based triazole hybrids 146a–b.

1,2,4-Triazole-maleamic acid derivatives 146a–b showed profound action against carbonic anhydrases, one of the main oncogenic targets. Compounds 146a–b were synthesized by reacting guanidine hydrochloride 142 with hydrazine hydrate in dioxane at 102 °C for 4 h to give triaminoguanidine hydrochloride 143 which was further reacted with different aryl aldehydes 144 to form tris-Schiff's bases 145. At the final stage, maleic anhydride was made to react with the pre-formed tris-Schiff's bases 145 in the presence of triethylamine to produce final target compounds 146a–b in good yields. Compound 146a showed good IC_50_ values of 124.22 and 114.83 µM against human carbonic anhydrase (hCA I) whereas compound 146b showed IC_50_ values of 120.57 and 95.17 µM against hCA I and hCA II when compared to standard carbonic anhydrase inhibitor acetazolamide. Docking studies displayed prominent interactions including H-bonding with Phe534, Asn62 and His64 along with arene–cation interaction with Arg516 (Scheme 23).^122^

**Scheme 24:**
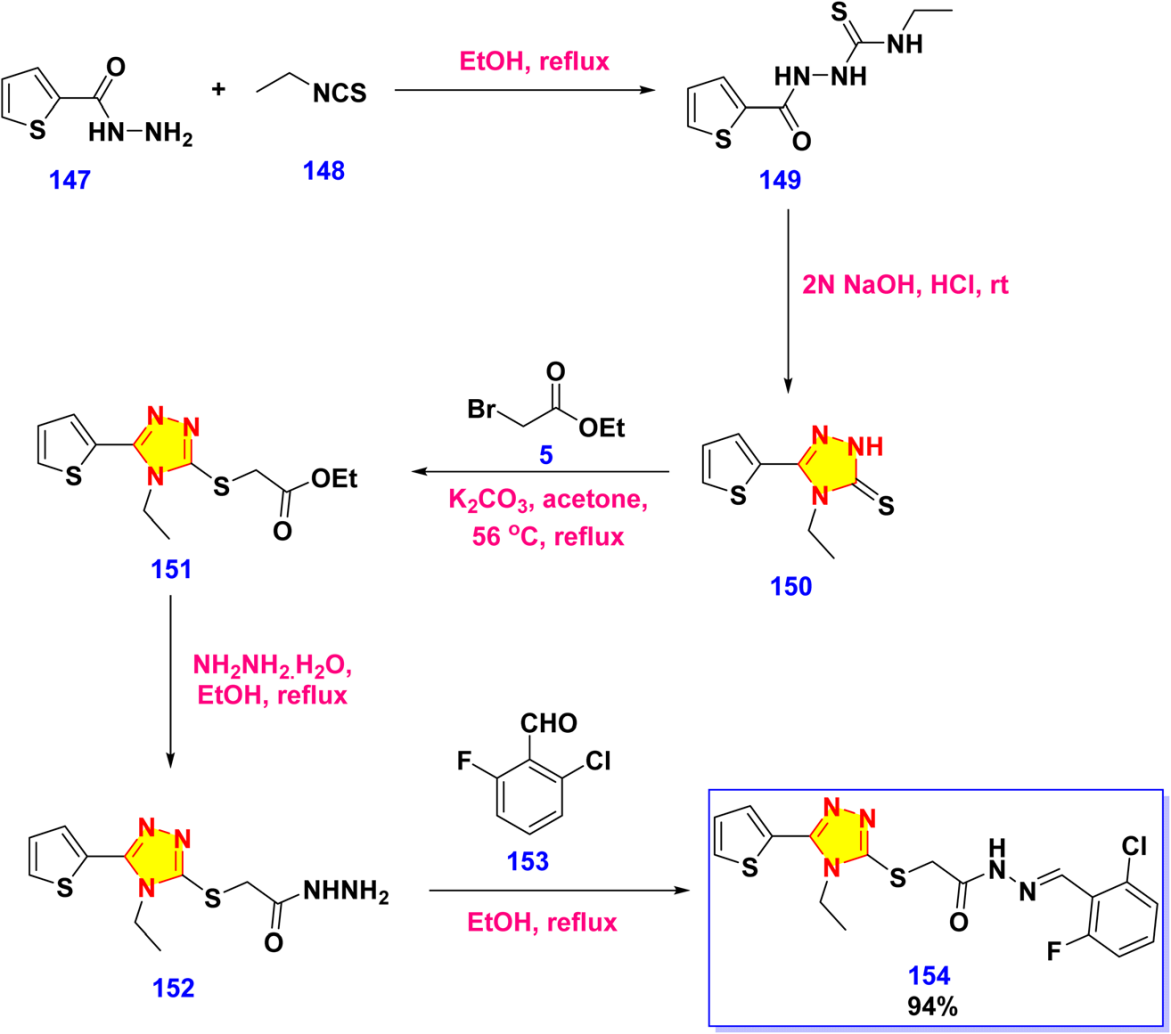
Synthesis of thiophene-triazole linked Schiff's base 154.

Thiophene-triazole linked Schiff's bases were observed to be excellent in their action. Likewise, compound 154 also showed promising effects against carbonic anhydrases targeting antiproliferative routes. This compound 154 was prepared from thiophene hydrazide 147 reacting with 4-ethylisothiocyanate 148 to generate thiosemicarbazide moiety 149 which was later cyclized to 1,2,4-triazole 150 using 2N NaOH in HCl at room temperature. This cyclized intermediate 150 was treated with ethyl-2-bromoacetate 5 under reflux conditions to give *S*-alkylated ester 151 which was subsequently converted to corresponding hydrazide 152 using hydrazine hydrate in ethanol under reflux. Finally, 2-chloro-6-fluorobenzaldehyde 153 was added to the pre-final compound 152 to give bioactive carbonic anhydrase inhibitor 154 in 94% yield. This compound 154 showed possessed IC_50_ value of 17.33 and 13.07 µM against hCA I and hCA II compared to acetazolamide. Molecular docking illustrated major interactions such as H-bonding with Phe534 and hydrophobic contacts with Val415, Ala493, Leu491, Leu490 (Scheme 24).^123^

**Scheme 25:**
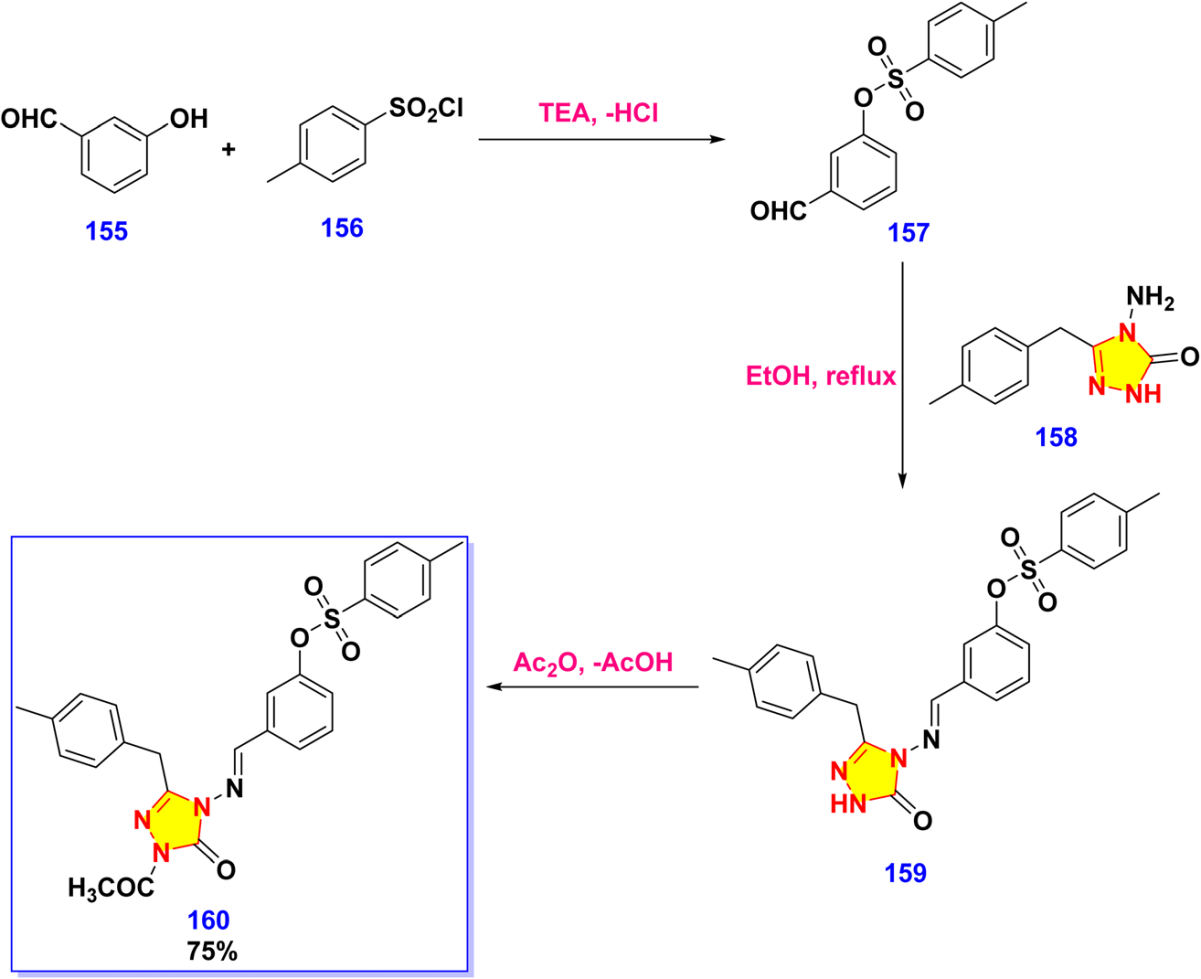
Synthesis of triazole-linked sulfonyl carbonic anhydrase inhibitor 160.

Triazole-linked sulfonyl compound 160 was constructed from 3-hydroxybenzaldehyde 155 and 4-methylbenzenesulfonyl chloride 156 in triethylamine which gives a condensed product 157 and further treatment with 1,2,4-triazole amine derivative 158 furnished Schiff's base 159. *N*-acetylation of 1,2,4-triazole motif 159 was carried out in acetic anhydride which eliminates acetic acid as a side product and accomplishes active carbonic anhydrase inhibitor 160 in high yield. This compound 160 was active against carbonic anhydrases with IC_50_ values of 7.12 and 10.62 µM against hCA I and hCA II, respectively compared to acetazolamide. Major interactions included H-bonding with Thr200. Alongside, there were many hydrophobic contacts with Leu60, Phe131, Ile91, Leu198, Val143, Ala142, Leu141 (Scheme 25).^124^

**Scheme 26:**
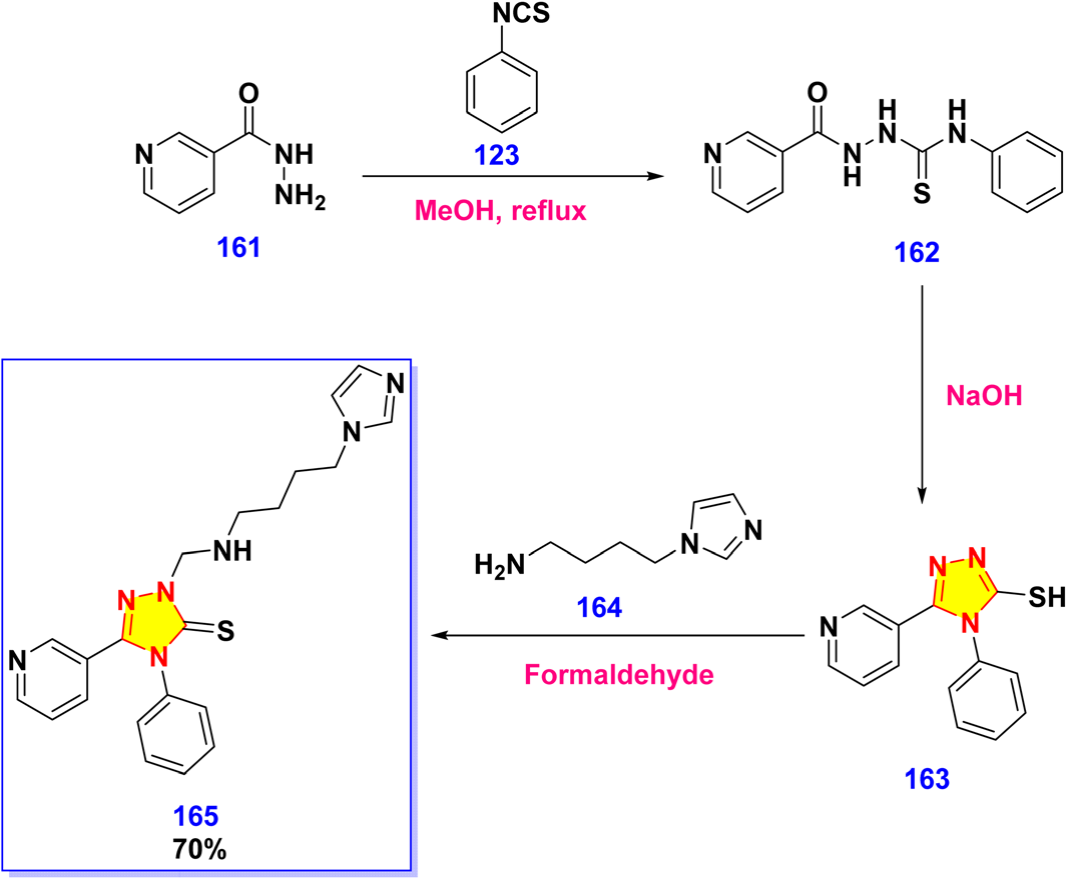
Synthesis of pyridine-containing 1,2,4-triazole hybrid 165.

Pyridine-containing triazoles have shown good effects on anticancer activity as we have seen in EGFR and VEGFR-2 inhibition. Furthermore, they are found to be effective against carbonic anhydrases with expanded activity. Compound 165 was furnished from the synthetic route starting from formation of thiosemicarbazide aromatic moiety 162 from pyridine-3-hydrazide 161 and phenylisothiocyanate 123 and later cyclized to form 1,2,4-triazole thiol 163 which was then tautomerized to keto form and combined with an aliphatic imidazole amine 164 in formaldehyde to give compound 165. This compound 165 reflected good action against carbonic anhydrase hCA I with IC_50_ value of 0.346 mM compared to acetazolamide. Docking analysis showed interactions such as H-bonding with Asn11, His64 and Glu239. Hydrophobic interactions with Leu198, Val121 and Ala65 (Scheme 26).^125^

**Scheme 27:**
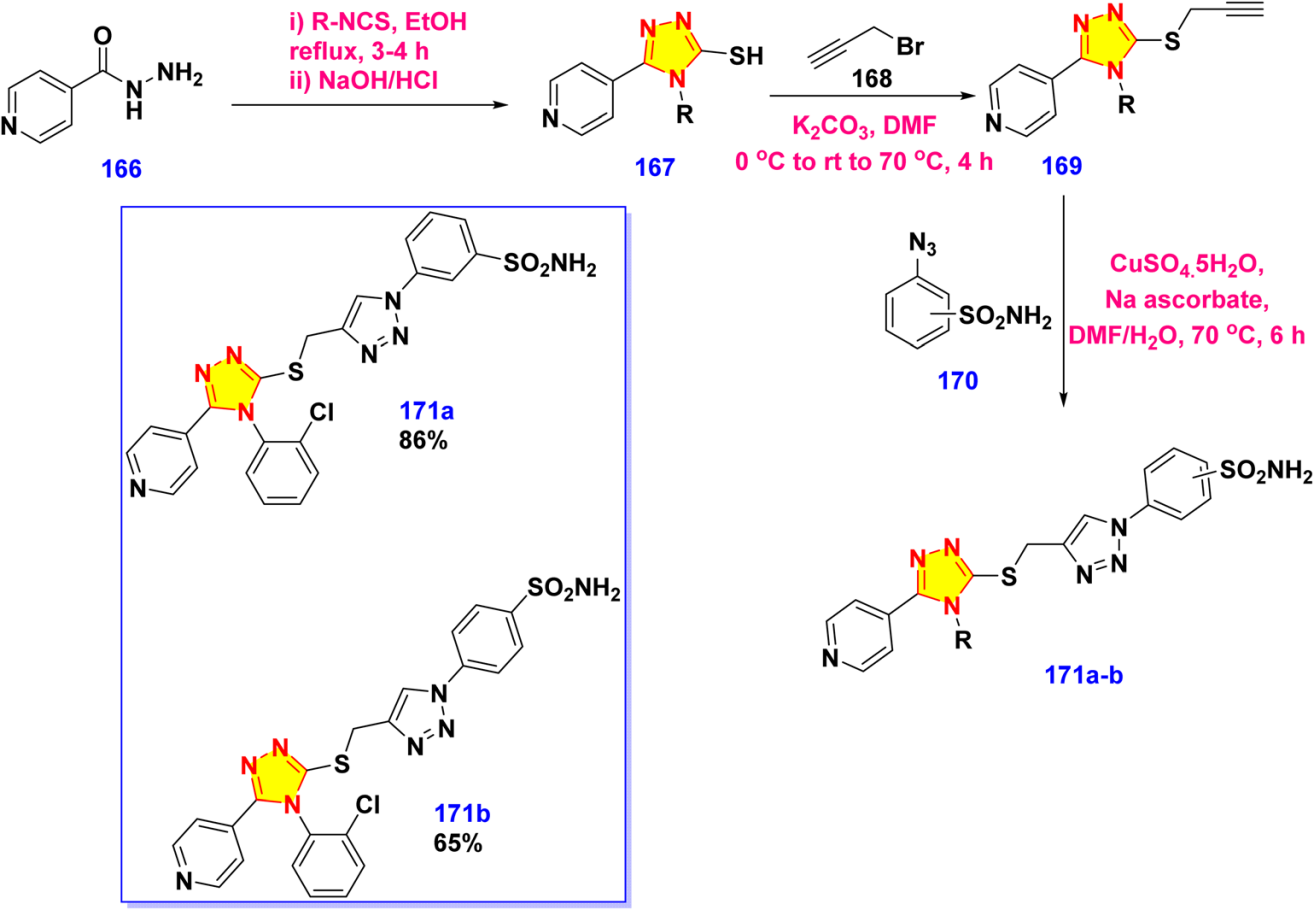
Synthesis of pyridine-containing 1,2,4-triazole sulfonamide analogues 171a–b.

As seen previously, pyridine-containing trialogues like compounds 171a–b showcased inhibitory action against carbonic anhydrases which were designed and constructed from pyridine-4-hydrazide 166 with different isothiocyanates in ethanol and refluxed for 3 and 4 h and subsequent ring formation 167 using NaOH in HCl. Later, propargyl bromide 168 was used to propargylate thiol group followed by copper-catalyzed alkyne–azide cycloaddition using copper sulfate pentahydrate in sodium ascorbate reacting alkyne substrate 169 with different sulfonamides 170 to achieve final compounds 171a–b in appreciable yields. These compounds 171a highlighted significant carbonic anhydrase inhibition with IC_50_ values of 25.1 and 35.2 nM against hCA IX and hCA X whereas compound 171b possessed IC_50_ values of 28.1 and 11.6 nM against the above carbonic anhydrases. As we have seen in EGFR inhibition by triazole derivatives, 1,2,4-triazole when linked with 1,2,3-triazole connected with phenyl sulfonamide garners significant attention due to the robust stability offered by 1,2,4-triazole and metabolic linker 1,2,3-triazole connected with sulfur-linkage for the improved carbonic anhydrase inhibition. Molecular interactions involved H-bonding with Thr200 and π–alkyl interaction with Leu91, Val121 and Gln92 (Scheme 27).^126^Table 4 presents an overview of the so far discussed 1,2,4-triazole containing carbonic anhydrase inhibitors.

**Table 4:** Overview of 1,2,4-triazole-containing carbonic anhydrase inhibitors

Compounds	Substituents	Major interactions	SAR observations
146a	*N*,*N*-Dimethylphenyl rings attached to triazole ring	H-bonding with Phe534. Arene–cation interaction with Arg516	Methyl groups attached to nitrogen atom increased cytotoxic effect
146b	3,4-Dichlorophenyl rings attached to triazole ring	H-bonding with Asn62 and His64. Arene–cation interaction with Arg516	Dichloro groups significantly enhanced phenyl rings' biological activity
154	2-Chloro-6-fluorobenzaldehyde linked to triazole moiety	H-bonding with Phe534. Hydrophobic contacts with Val415, Ala493, Leu491, Leu490	Both chloro and fluoro groups on the phenyl ring system increased carbonic anhydrase inhibition by occupying the binding pocket of hCA I and hCA II
160	4-Tolyl group linked through methylene bridge to triazole	H-bonding with Thr200. Hydrophobic contacts with Leu60, Phe131, Ile91, Leu198, Val143, Ala142, Leu141	Electron donating methyl group increased cytotoxicity with anticancer effect
165	Pyridine and phenyl rings connected to triazole hybrid	H-bonding with Asn11, His64 and Glu239. Hydrophobic interactions with Leu198, Val121 and Ala65	Two electron-dense aromatic ring systems occupied the hinge region of the carbonic anhydrase II increasing binding affinity
171a–b	2-Chlorophenyl rings attached to triazole analogue	H-bonding with Thr200. Alkyl interaction with Leu91, Val121 and Gln92	Electron-withdrawing chlorophenyl group improved cytotoxicity

## 3 Conclusion and future outlook

From this review, it is clear that 1,2,4-triazoles have a significant and tremendous scope in the field of medicinal chemistry and oncogenic studies. Most of the compounds discussed in the review have been potent in terms of their initial anticancer profiles and studies compared to the standard reference drugs. Most often, Schiff's bases when linked with 1,2,4-triaozle hybrids, increased the rate of inhibition. Different aromatic systems linked with 1,2,4-triazoles present different kinds of profiles with positive signs of emerging as anticancer agents. When triazoles are connected with a few more five-membered heterocyclic motifs such as pyrazole, imidazole, and thiazole, they develop biological action and inhibit tyrosine kinases. When 1,2,4-triazoles are fused within five-or six-membered cyclic systems, their activity increases, with good cytotoxic profiles and anticancer effects. Molecular docking studies revealed the major interactions responsible for the enhanced biological performance of the bioactive compounds. Various interactions include H-bonding, π–π stacking, π–alkyl interactions, hydrophobic contacts, π–sigma interactions, van der Waals interactions, arene–cation interactions. SAR observations help us understand the effects of various substituents linked to 1,2,4-triazole moieties either directly or through different bonds. Understanding the influence of substituents on the activity of compounds with respect to the development of anticancer agents is very important.

This review highlights the recent trends in 1,2,4-triazole chemistry which is equally important as that of biological research. By understanding the in-depth synthetic chemistry aspects of how triazole rings are produced using different methods, it is possible for chemists to develop new triazole scaffolds with ease. Therefore, most of the synthetic routes explored throughout this review show diverse synthetic uses in the production of biologically active 1,2,4-triazole analogues. Most of the compounds covered in this review have shown better results than do the commercially available well-known anticancer drugs such as Erlotinib, Sorafenib, Sunitinib, Gefitinib, Acetazolamide and many more. Among the three isomeric forms of 1,2,4-triazoles, 1*H*-1,2,4-triazole remains to be the most active isomeric form with high thermodynamic stability and good receptor interaction offering significant results with respect to anticancer activity followed by 4*H*-1,2,4-triazole core derivatives. Thus, the scope of triazole chemistry research has widened with prominent and promising results displayed through anticancer activities.

In the upcoming years, addressing the current challenges faced by chemists and biologists in the production of new drug molecules which include failure in the last phases of oral bioavailability, clinical trials and dosage compensation procedures, is necessary. Therefore, green chemistry could be favorable for chemists to work on 1,2,4-triazole chemistry, which garners wide applicability in the field of synthetic chemistry. Most of the bioactive compounds discussed in this review are produced in good to high yields whereas few molecules whose yields have been low and it is better for us to address this challenge associated with synthetic chemistry and find new reaction routes for the efficient synthesis of 1,2,4-triazoles.

## Ethical statement

This study does not involve the use of any humans or animals.

## Author contributions

Glanish Jude Martis: writing – original draft, software; Eshitha Jane D Souza: writing – original draft, software; Praveen S. Mugali: writing – review and editing; Santosh L. Gaonkar: writing – review and editing, supervision.

## Conflicts of interest

The authors declare no competing financial interests.

## Data Availability

All data has been obtained from peer-reviewed articles cited in the reference list, with no additional datasets utilized.
